# Biomarkers of Inflammation and Axonal Degeneration/Damage in Patients with Newly Diagnosed Multiple Sclerosis: Contributions of the Soluble CD163 CSF/Serum Ratio to a Biomarker Panel

**DOI:** 10.1371/journal.pone.0119681

**Published:** 2015-04-10

**Authors:** Morten Stilund, Mikkel Carstensen Gjelstrup, Thor Petersen, Holger Jon Møller, Peter Vestergaard Rasmussen, Tove Christensen

**Affiliations:** 1 Department of Neurology, Aarhus University Hospital, Nørrebrogade 44, DK-8000 Aarhus C, Denmark; 2 Department of Clinical Biochemistry, Aarhus University Hospital, Nørrebrogade 44, DK-8000 Aarhus C, Denmark; 3 Department of Biomedicine, Bartholin Building, Wilhelm Meyers Allé 4, Aarhus University, DK-8000 Aarhus C, Denmark; Medical University of Innsbruck, AUSTRIA

## Abstract

**Background:**

Expression of soluble CD163 (sCD163), a macrophage/microglia biomarker, is increased in inflammatory conditions, and sCD163 levels in the cerebrospinal fluid (CSF) have recently been shown to be elevated in patients with multiple sclerosis (MS): the sCD163 CSF/serum ratio was elevated in patients with relapsing-remitting MS (RRMS), primary progressive MS (PPMS), and clinically isolated syndrome (CIS) compared with symptomatic controls.

**Objective:**

To investigate the contributions of the sCD163 CSF/serum ratio to a biomarker panel focusing on inflammation and axonal degeneration in newly diagnosed MS; thus optimising a diagnostic biomarker panel for MS.

**Methods:**

After a full MS diagnostic work-up, including collection of paired samples of CSF and serum, 125 patients were included in this study. Patients were divided into groups based on their diagnosis, and patients with normal clinical and paraclinical findings were defined as symptomatic controls. Serum and CSF levels, ratios, and indices of sCD163, CXCL13, osteopontin, neopterin, and CSF levels of neurofilament light polypeptide were determined by enzyme-linked immunosorbent assays (ELISAs). For sCD163 the results constitute a post-hoc analysis of already published data.

**Results:**

All tested biomarkers, notably the sCD163 ratio, the CXCL13 ratio, the NEO ratio, the CSF level of NfL, the IgG index, and the serum level of OPN, were significantly correlated to RRMS, PPMS, and/or CIS. The individual biomarkers in single tests had a lower performance than the IgG index, however, their combined receiver operating characteristic (ROC) curve demonstrated excellent diagnostic discriminatory power.

**Conclusion:**

The biomarker panel showed distinct profiles for each patient group and could be a valuable tool for clinical differentiation of MS subgroups. The combined ROC analysis showed that sCD163 contributes positively as a diagnostic marker to a panel of established MS biomarkers. Patients with PPMS were demonstrated to have significantly elevated levels of both inflammatory and degenerative markers.

## Introduction

Multiple sclerosis (MS) is the most common demyelinating disease of the central nervous system (CNS) in young adults. The cause of the disease is unknown, yet it is generally assumed to be complex interactions between environmental factors and genetic susceptibility [1–3]. At the time of diagnosis, the disease course of patients with MS is characterized by either being remitting-relapsing (RRMS) or progressive: primary progressive MS (PPMS)—or in some cases with previous attacks, secondary progressive MS (SPMS). Patients with clinically isolated syndrome (CIS) often progresses to MS (approximately 85%) [4]. Currently, MS diagnostic workup consists of an anamnestic support for spreading of neurological symptoms in time and space, MRI findings, and CSF/serum biomarkers such as an increased IgG index. A pathognomonic biomarker for MS has never been identified, yet substantial effort has been put into testing candidate molecules in various body fluids from patients. Several different molecules have been suggested as potential diagnostic biomarkers, but they still need to be validated in patient cohorts [5–9]. As suggested in previous reviews, the research in the field of MS biomarkers should be directed towards earlier and more accurate diagnosis to initiate disease modifying therapy [10–12].

A characteristic feature of MS lesions (plaques) is the abundant presence of macrophages in all types (I-IV) of MS lesions [13]. Macrophages are components of the innate immune system and as tissue specific cells in the CNS, their functions are multifaceted, ranging from pro-inflammatory and antigen presenting (M1 pathway) to anti-inflammatory and growth promoting (M2 pathway) [14, 15]. Both pro- and anti- inflammatory macrophages as well as intermediate stages have been shown to be present in active MS lesions [16, 17].

**Fig 1:**
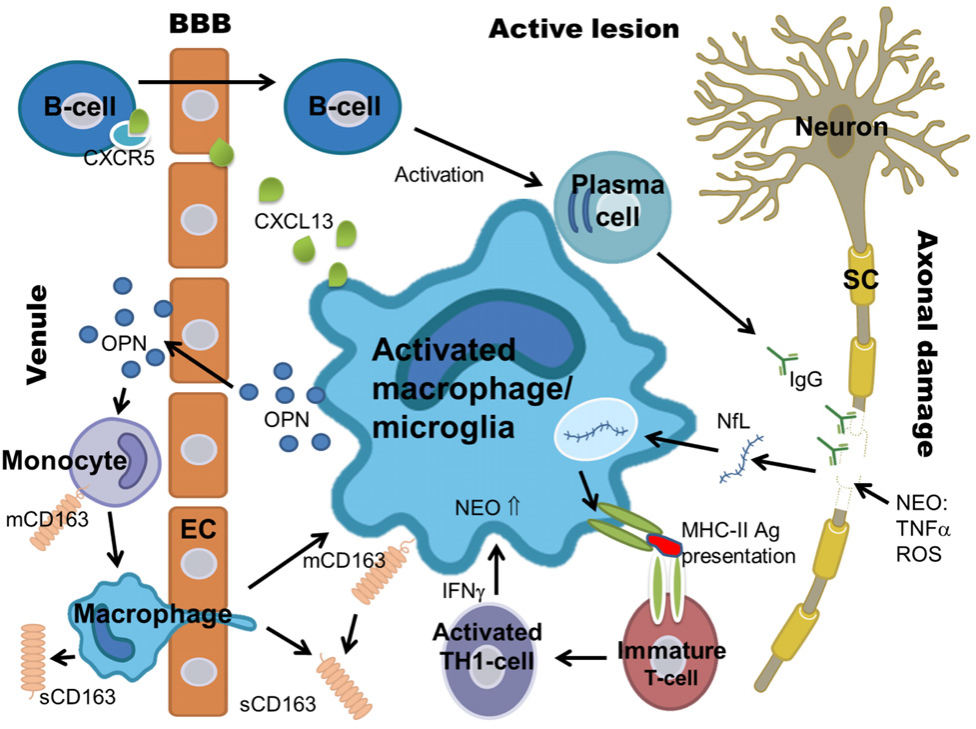
Macrophages and microglia and the suggested roles of sCD163, CXCL13, NEO, NfL, OPN, and IgG in MS pathology. Fig 1 shows the biomarkers sCD163, CXCL13, NEO, NfL and OPN and their relation to monocyte activation, macrophage migration, and interactions with macrophages/microglia in active (acute/chronic) MS lesions. Biomarker characteristics are listed below: Abbreviations: sCD163 (soluble CD163), mCD163 (membrane bound CD163), NEO (neopterin), NfL (neurofilament light polypeptide), OPN (osteopontin), Th-cells (T helper cells), MS (multiple sclerosis), CSF (cerebrospinal fluid), EC (epithelial cell), Ag (antigen), SC (Schwann cell), OND (other neurological disease), ROS (reactive oxygen species).

We have recently demonstrated a significant association between levels of the sCD163 CSF/serum ratio (sCD163 ratio) and MS/CIS [18]. Here, in the same patient cohort, we present further analyses on serum and CSF to evaluate the diagnostic contributions of sCD163 and the sCD163 ratio to a panel of established biomarkers focusing on inflammation and axonal damage. The biomarkers chosen for the present study were sCD163, CXCL13, neopterin (NEO), neurofilament light polypeptide (NfL), and osteopontin (OPN). This selection of biomarkers was based on previously published reports [9, 17–54] using the following criteria: 1) known MS diagnostic properties, 2) association with macrophage activity, and 3) detectability in CSF and/or serum by means of a previously validated quantifying method. Biomarkers and their characteristics are listed in table 1 and shown in Fig 1.

**Table 1:** Biomarkers and their characteristics.

Biomarker	Molecule specifics	Know pathology	Known/Suggested function in MS	References
**sCD163**	SRCR protein family. Membranous form (mCD163) is a receptor for haptoglobin—hemoglobin complexes.	Serum levels of sCD163 are elevated in macrophage activation syndrome, sepsis, and liver disease. sCD163 is shed by monocytes in serum. In the CNS sCD163 may arise from macrophages and microglia.	sCD163 may be related to bacterial binding and anti-inflammatory action. Is a macrophage activation marker. Increased levels in CSF of MS patients.	[17–22]
**CXCL13**	CXCL13 is a chemokine in the CXC family. Its ligand is CXCR5 (BLR-1) which is present mainly on B-cells but also on T-cells and monocytes/macrophages.	B-cell recruitment to the CSF in the event of neuroinflammatory conditions. Marker of inflammation and increased MS disease activity. Elevated CXCL13 levels have been shown in RRMS, PPMS, and CIS disease courses.	Levels of CXCL13 are correlated to the amount of CSF B-cells, plasmablasts, and intrathecal Ig production. CXCL13 is produced by follicular dendritic cells, however in inflammatory conditions the chemokine is mainly expressed by monocytes/macrophages / microglia.	[23–34]
**NEO**	NEO is a purine nucleotide belonging to the pteridines and it is produced by monocytes and macrophages when stimulated by IFN-gamma	In chronic inflammation the release of NEO contributes to an environment of inflammatory mediators such as TNF-alpha, nitric oxide (NO) and hydrogen peroxide (H2O2) and feed-back mechanisms facilitates a milieu of oxidative stress.	Elevated levels of NEO have been found in serum and CSF in several studies of MS and the NEO CSF/serum ratio (NEO ratio) has also been found significantly elevated in patients with RRMS compared with healthy controls.	[35– 41]
**NfL**	NfL is one of three different protein chains that constitutes the axonal cytoskeleton	When axonal damage occur neurofilaments are released into the extracellular fluid and can be measured in the CSF as an indication of early acute axonal damage	Macrophages and microglia phagocytises neurofilament. Antigens in MS lesions are presented by macrophages and microglia to Th-cells and mediate development of inflammatory responses towards the neurons.	[42–49]
**OPN**	OPN is a phosphorylated protein secreted by macrophages, T-cells and epithelial cells into most body fluids and it has a plethora of binding-sites where lymphocyte binding and chemokine induction are key features.	OPN enhances phagocytosis Activated monocytes/ macrophages uses OPN as a chemotactic agent and as monocytes and macrophages express high levels of integrins that binds OPN, this is vital for macrophage migration.	OPN is upregulated in the CNS of patients with MS and patients with OND. OPN levels in plasma have been suggested as a biomarker of disease activity; however, a recent study could not support this.	[50–54]

Table 1 shows the biomarkers analyzed in the study and their characteristics. Abbreviations: sCD163 (soluble CD163), mCD163 (membrane bound CD163), NEO (neopterin), NfL (neurofilament light polypeptide), OPN (osteopontin), Th-cells (T helper cells), MS (multiple sclerosis), CSF (cerebrospinal fluid).

## Objectives

The objectives of the present study were to investigate the contributions of the sCD163 ratio to a biomarker panel focusing on inflammation and axonal degeneration in patients with newly diagnosed MS. The study was performed on a large cohort of patients diagnosed with remitting relapsing MS (RRMS), primary progressive MS (PPMS), or clinically isolated syndrome (CIS). All cases were compared with a group of symptomatic controls (SC) [55]. The applicability of these biomarkers in MS diagnostics, individually and collectively, was evaluated.

## Materials and Methods

### Ethics statement

The study was conducted in accordance with the Ethical Declaration of Helsinki and all patients gave written, informed consent. The study and the material for informed consent were approved by The Central Denmark Region Committee on Biomedical Research Ethics (journal number: 20090210).

### Patient cohort

Patients admitted for further diagnostic investigations to the MS clinic, Department of Neurology, Aarhus University Hospital were consecutively included in the study during a period from September 2009 to December 2011. A full diagnostic work-up included medical history, clinical examination, MRI of the entire neural axis, CSF analyses (cells, protein, immunoglobulin G (IgG) index), and evoked potentials as recommended [56]. CSF and MRI examination were evaluated according to the revised MacDonald criteria [56] and an EDSS (extended disability status scale) score was assessed according to Kurtzke [57]. Mean total number of MRI white matter lesions (presented in table 2) were registered by fluid-attenuated inversion recovery (FLAIR) sequences on MRI.

**Table 2:** Demographic and clinical data of the patients with MS/CIS and SC.

Characteristics	RRMS	PPMS	CIS	SC
**No. of Subjects (total = 125)** ^**a**^	n = 44	n = 15	n = 27	n = 39
**Gender M/F**	7/37	8/7	7/20	4/35
**Mean Age** ^**b**^	37	53	37	41
**(range)**	(23–62)	(35–64)	(16–71)	(25–57)
**Mean EDSS**	2.5	3.7	1.7	0
**(range)**	(0–4.0)	(2.0–7.0)	(0–3.0)	N/A
**Mean No. of attacks** ^**c**^	2.91	0	1	N/A
**Disease Duration**	57.4	55	18.4	0
**(range)** ^**d**^	(3.5–288)	(14–228)	(0.5–90)	N/A
**Time since last attack (d)**	131	N/A	388	N/A
**(range)** ^**e**^	(1–720)	N/A	(3–2745)	N/A
**Mean CSF Protein, μmol/L**	0.38	0.41	0.39	0.33
**(range)**	(0.2–0.63)	(0.2 1–0.65)	(0.16–0.74)	(0.20–0.57)
**Mean CSF Cells, 10^6/L**	8.8	4.6	6.0	1.6
**(range)**	(0–39)	(1–12)	(0–55)	(0–5)
**Mean IgG Index**	1.20	1.09	0.89	0.49
**(range)**	(0.44–3.04)	(0.44–3.94)	(0.42–2.81)	(0.40–0.62)
**Mean total number of MRI white matter lesions**	18	18	9	0
**(range)**	(4–55)	(2–45)	(0–42)	N/A

Table 2 shows the demographics and clinical data of the patient cohort. These demographic and clinical data are essentially as previously presented [18].

^a^Refers to the patients included.

^b^Age (in years) refers to age at the time of sampling.

^c^Mean number of attacks: mean number of attacks before the sampling time point.

^d^Disease duration (in months): the period of time from debut of first symptom(s) to the sampling time point.

^e^Time since last attack: the period of time (in days) from latest attack to the sampling time point.

Mean total number of MRI white matter lesions were registered by fluid-attenuated inversion recovery (FLAIR) sequences on MRI. Abbreviations: RRMS (relapsing-remitting MS), PPMS (primary-progressive MS), CIS (clinically isolated syndrome), SC (symptomatic controls), N/A (not applicable or available), n (number of persons), EDSS (Expanded Disability Status Scale), CSF (cerebrospinal fluid).

In total 183 patients agreed to participate in this study and in accordance with consensus guidelines [58] serum and CSF samples were collected and frozen at -70°C. After aliquotation, all analyses were performed immediately after thawing of the aliquots and no samples were subjected to further freeze-thaw cycles. Demographics and paraclinical findings are summarised in table 2.

This patient cohort was also the cohort in the recent study of sCD163 levels and macrophage activity in MS [18]. In the present study five patients from the original cohort were excluded: four patients with SPMS (as these were too few to be representative for this MS subgroup) and one patient with RRMS (due to lack of sample material). None of the patients in the cohort received methylprednisolone or other immune-modulating therapeutics for at least a month before the time of sampling.

### ELISA analyses

Levels of sCD163 were analysed by ELISA as previously described [59]. In brief, rabbit anti-CD163 (2 mg/L, SK Moestrup Aarhus University) was coated into microtitre wells and serum samples (diluted 1:101) or CSF samples (diluted 1:4) were added and incubated for 1 h at RT. Monoclonal anti-CD163 (clone number: GHI/61.3 μg/mL) was added followed by incubation for 1 h at RT with horseradish peroxidase-labelled goat anti-mouse antibodies (0.125 μg/mL; Dako, Glostrup, Denmark).

Levels of CXCL13, NEO, and OPN were analysed in both serum and CSF and levels of NfL were only analysed in CSF by enzyme-linked immunosorbent assay (ELISA) following the instructions given by the manufacturers. The available kits for NfL at the time of analysis were restricted to be used only with CSF. Samples were run in duplicates and a coefficient of variation (CV) was calculated, accepting only values ≤15%. Intra assay variations were calculated from six individual measurements of a known standard on each plate and values ≤15% were accepted (ranges: CXCL13 (2.02–8.57), NEO (3.54–13.67), NF-Light (0.58–5.94%), and OPN (2.00–9.80%)). Samples with values exceeding the highest point of the standard curve were diluted and reanalysed. The diagnoses were established before the results of the sCD163 analyses were received. For the other four biomarkers each plate contained 36 randomly selected samples, and each sample were labelled with a study ID and assayed in a manner blinded to the clinical status of the patients.

CXCL13 levels (R&D Systems, Minneapolis, DCX130) were measured in a competitive ELISA assay by adding 100 μL assay diluent and 50 μL of sample to each well, followed by a two hour incubation period. After 4 × wash, 200 μL of conjugate (mAb-HPR) were added, followed by two hours incubation. After 4 × wash, 200 μL substrate solution (TMB) were added and the plate was incubated for 30 min protected from light. Finally, 50 μL of stop solution (H_2_SO_4_) were added and the absorbance was measured on an ELISA plate reader (Thermo Scientific, Multiscan FC) at 450 and 540 nm. Concentrations were calculated by linear regression in Excel.

NEO levels (IBL Int. GMBH, Hamburg, RE59321) were measured by adding 100 μL enzyme conjugate (NEO), 20 μL of sample and 50 μL of NEO antiserum (pAb-HPR) to each well, followed by a 90 min incubation period. After 4 × wash, 150 μL of substrate solution (TMB) were added and the plate was incubated for 10 min protected from light. Finally, 150 μL of stop solution (H_2_SO_4_) were added and the absorbance was measured on an ELISA plate reader (Thermo Scientific, Multiscan FC) at 450 and 620 nm. Concentrations were calculated by 4-parametric statistical regression in GraphPad Prism.

NfL (NF-light) (UmanDiagnostics, Umea, UD51001) levels were measured by adding 50 μL sample dilution buffer and 50 μL of sample to each well, followed by one hour incubation. After 3 × wash, 100 μL of tracer (mAb) were added, followed by 45 min incubation. After 3 × wash, 100 μL conjugate (HPR) were added and the plate was incubated for 30 min. After 3 × wash, 100 μL substrate solution (TMB) were added and the plate was incubated for 15 min protected from light. Finally, 50 μL of stop solution (H_2_SO_4_) were added and the absorbance was measured on an ELISA plate reader (Thermo Scientific, Multiscan FC) at 450 and 540 nm. Concentrations were calculated by 4-parametric statistical regression in GraphPad Prism.

OPN levels (R&D Systems, Minneapolis, DOST00) were measured by adding 100 μL assay diluent and 50 μL of sample (diluted 1:25) to each well, followed by a two hour incubation period.

After 4 × wash, 200 μL of conjugate (pAb-HPR) were added, followed by two hours incubation. After 4 × wash, 200 μL substrate solution (TMB) were added and the plate was incubated for 30 min protected from light. Finally, 50 μL of stop solution (H_2_SO_4_) were added and the absorbance was measured on an ELISA plate reader (Thermo Scientific, Multiscan FC) at 450 and 540 nm. Concentrations were calculated by 4-parametric statistical regression in GraphPad Prism.

The manufacturer comments that OPN levels in serum are validated but may be reduced as a consequence of proteolytic cleavage during clotting.

### Collection of data and statistical analysis

Data were stored and handled according to the Danish law on personal data. During collection of demographic and biochemical data we used the Electronic Patient Journal (EPJ). Descriptions of MRI data was conducted by a neuro-radiologist and confirmed by a senior neurologist who viewed all scans in the IMPAX system at the Department of Neurology, AUH.

For data collection we used Microsoft Excel and all statistical analysis was performed using STATA12. Please see Figure A in S1 Dataset for full data. Spearman correlation was used to correlate variables that were on an ordinal scale and Pearson pairwise correlation analyses were used to correlate continuous variables. This was done to identify, firstly, biomarkers that were correlated to age and or gender so that these could be adjusted for in our regression analysis. Secondly, correlations were performed to identify whether biomarkers were correlated to CIS/MS groups and thirdly, whether there was any biomarker intercorrelation. Please see tables B—AA in S1 Dataset for STATA coding and output. Since biomarkers in CSF and serum were positively skewed, we performed correlation and regression analyses on LOG-transformed data, except the intercorrelation analysis that was performed without the LOG-transformation. Only biomarkers that correlated significantly to CIS, RRMS or PPMS were included in the regression analysis, table AB in S1 Dataset. The geometric means (GM) of the SC group were used as baseline and the difference is presented in fold with 95% confidence intervals (95%CI) and p-values [60].

For calculations of ratios (i.e. sCD163 ratio) the CSF concentration was divided by the serum concentration, and indices were calculated by dividing the biomarker CSF/serum ratio by the albumin ratio.

The receiver operating characteristic (ROC) analyses were performed for the variables that were most significantly different from the SC group in the regression analysis. The parameter results for patients with RRMS, PPMS, and CIS are combined as true positives and plotted against SC as true negatives. A logistic regression analysis was performed for the combined diagnostic properties for each biomarker and the IgG index, and the entire biomarker panel including the IgG index [61]. For a view on the data generated please see tables AC and AD in S1 Dataset for STATA do-files and output.

## Results

### sCD163

**Fig 2:**
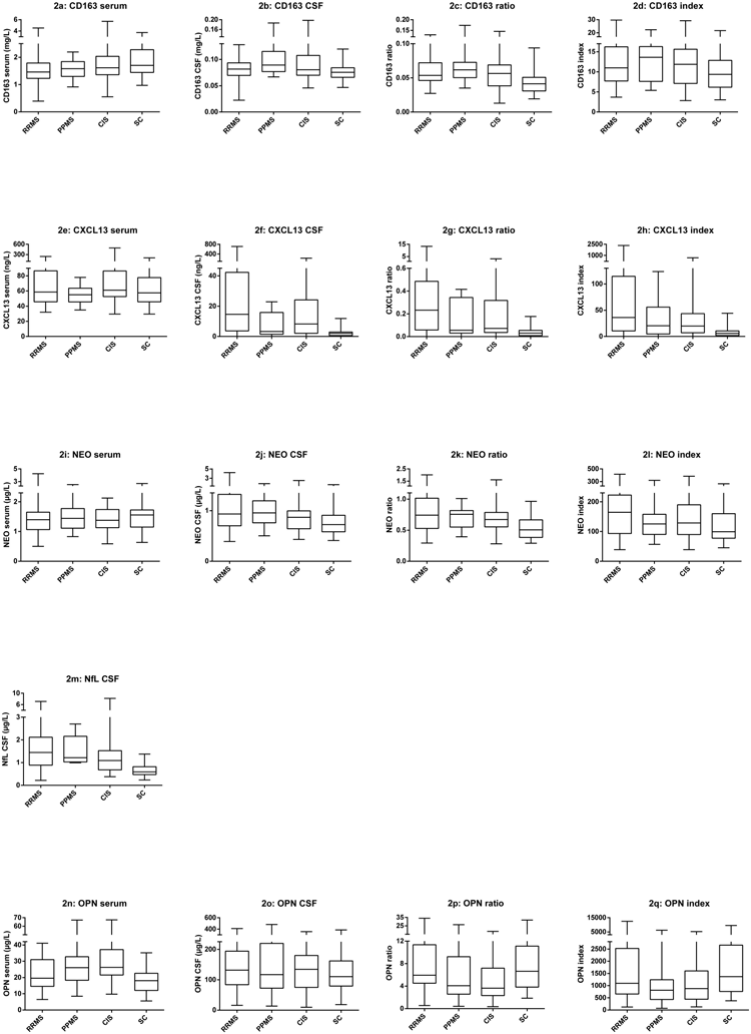
Box plots of sCD163, CXCL13, NEO, NfL, and OPN. Fig 2 shows box plots of all biomarkers including CSF and serum concentrations as well as ratios and index values. The box plots show levels of all the parameters for each group, RRMS, PPMS, CIS and SC. The horizontal line in each box is the median, whiskers fences the upper and lower quartiles. The data on sCD163 have previously been presented in [18]. Fig 2a–2d shows the values for sCD163, Fig 2e–2h shows the values for CXCL13, Fig 2i–2l shows the values for NEO, Fig 2m shows the values for NfL CSF, and Fig 2n–2qshows the values for OPN. For all biomarkers, there were some overlaps in levels between patient groups, and symptomatic controls. Abbreviations: RRMS (relapsing-remitting MS), PPMS (primary-progressive MS), CIS (clinically isolated syndrome), SC (symptomatic controls), CSF (cerebrospinal fluid), sCD163 (soluble CD163), NEO (neopterin), NfL (neurofilament light polypeptide), OPN (osteopontin).

Soluble CD163 was detectable in CSF and serum from all individuals, as reported in [18]. The sCD163 levels, ratio and index values are presented in Fig 2a–2d. Median levels and ranges are also presented in table 3. Median serum levels of sCD163 for the patients with RRMS, PPMS, and CIS were somewhat lower than for the SC group. However, the sCD163 ratio was elevated, particularly for the patients with PPMS. For the SC group the median level of sCD163 in serum was 1.70 mg/L (range 0.97–3.73 mg/L). This corresponds well with the sCD163 serum reference interval for healthy subjects (0.7–3.9 mg/L) [21].

**Table 3:** Median levels and range of biomarkers in serum and CSF samples as determined by ELISA, for the patient groups.

Characteristics	RRMS	PPMS	CIS	SC
**No. of Subjects (total = 129)**	n = 44	n = 15	n = 27	n = 39
**Serum sCD163 mg/L**	1.45	1.58	1.62	1.70
**(range)**	(0.39–4.53)	(0.92–2.2)	(0.55–5.71)	(0.97–3.73)
**CSF sCD163 mg/L**	0.082	0.089	0.081	0.076
**(range)**	(0.023–0.128)	(0.067–0.187)	(0.046–0.197)	(0.047–0.120)
**sCD163 ratio**	0.054	0.062	0.057	0.041
**(range)**	(0.027–0.129)	(0.035–0.174)	(0.013–0.145)	(0.019–0.094)
**sCD163 index**	10.1	13.6	11.9	9.4
**(range)**	(3.7–29.7)	(5.4–22.4)	(2.9–29.2)	(3.0–21.7)
**Serum CXCL13 μg/L**	58.7	55.1	61.0	57.48
**(range)**	(32.1–242.1)	(34.9–77.8)	(29.6–493.9)	(29.5–201.4)
**CSF CXCL13 μg/L**	14.6	3.3	8.2	2.1
**(range)**	(0.0016–711.2)	(0.0016–22.9)	(0.0016–231.4)	(0.0016–11.8)
**CXCL13 ratio**	.233	0.051	0.072	.015
**(range)**	(0.00004–13.32)	(0.00002–0.41)	(0.00002–3.10)	(0.00002–0.18)
**CXCL13 index**	36.1	20.4	19.9	5.9
**(range)**	(0.0066–2343.7)	(0.0030–123.7)	(0.0027–678.8)	(0.0034–44.2)
**Serum NEO mg/L**	1.397	1.444	1.380	1.557
**(range)**	(0.501–4.245)	(0.831–2.529)	(.585–2.128)	(.634–2.696)
**CSF NEO mg/L**	.934	.956	.869	.724
**(range)**	(.390–4.223)	(.502–1.856)	(0.431–2.52)	(0.41–1.64)
**NEO ratio**	0.743	0.756	0.672	0.505
**(range)**	(0.293–2.014)	(0.393–1.010)	(0.280–1.607)	(0.29–0.964)
**NEO index**	165	126	128	99
**(range)**	(39–416)	(57–320)	(39–386)	(45–264)
**CSF NfL μg/L**	1.447	1.219	1.091	0.591
**(range)**	(0.218–7.123)	(0.996–2.7)	(0.38–8.209)	(0.233–1.376)
**Serum OPN μg/L**	19.54	26.06	26.23	18.05
**(range)**	(6.49–41.09)	(8.49–67.25)	(9.76–67.49)	(5.54–35.15)
**CSF OPN μg/L**	131.98	116.92	134.41	110.32
**(range)**	(15.93–409.97)	(13.53–482.21)	(9.48–356.70)	(18.49–387.53)
**OPN ratio**	5.94	4.07	3.64	6.64
**(range)**	(0.56–34.05)	(0.41–26.41)	(0.34–18.45)	(1.86–31.91)
**OPN index**	1094	810	1367	1072
**(range)**	125–12484	70–6213	127–5254	376–9572

Table 3 shows the median levels of sCD163, CXCL13, NEO, and OPN in serum, CSF, ratio, and index. Index formula: (Biomarker ratio / Albumin ratio). NfL has only been examined in CSF. Abbreviations: RRMS (relapsing-remitting MS), PPMS (primary-progressive MS), CIS (clinically isolated syndrome), SC (symptomatic controls), n (number of subjects), CSF (cerebrospinal fluid) sCD163 (soluble CD163), NEO (neopterin), NfL (neurofilament light polypeptide), OPN (osteopontin). The data on sCD163 have previously been presented in [18].

### CXCL13

CXCL13 was detectable in serum from all individuals, but CXCL13 in CSF was undetectable in samples from one patient each from the RRMS and CIS patient groups, respectively, and in samples from five patients from the SC group. For calculating statistics the lower detection limit of the assay (0.0016 μg/L) was used where concentrations of proteins were below the lower detection limit. The CXCL13 levels, ratio and index values are presented in Fig 2e–2h. Median levels and ranges are also presented in table 3. The CXCL13 levels in the CSF were highly elevated for the patients with RRMS (particularly), PPMS and CIS, compared with the SC group. For the SC group the median level of CXCL13 in serum was 57.48 μg/L (range 29.5–201.4 μg/L).

### Neopterin (NEO)

NEO was detectable in CSF and serum from all individuals. The NEO levels, ratio and index values are presented in Fig 2i–2l. Median levels and ranges are also presented in table 3. In a pattern similar to that of sCD163, median serum levels of NEO for the patients with RRMS, PPMS, and CIS were somewhat lower than for the SC group. However, the NEO ratio was elevated, particularly for the patients with PPMS. For the SC group the median level of NEO in serum was 1.56 μg/L (range 0.634–2.696 μg/L).

### Neurofilament light polypeptide (NfL)

NfL was detectable in CSF from all individuals. The NfL CSF levels are shown in Fig 2m. Median levels and ranges are also presented in table 3. The levels of NfL CSF were elevated for the patients with RRMS, PPMS and notably also for the patients with CIS, as compared with the SC group. For the SC group the median level of NfL CSF was 0.59 mg/L (range 0.233–1.376 μg/L).

### Osteopontin (OPN)

OPN was detectable in CSF and serum from all individuals. The OPN levels, ratio and index values are presented in Fig 2n–2q. Median levels and ranges are also presented in table 3. Interestingly, OPN levels in serum for the patients with RRMS, PPMS, and for the patients with CIS, were elevated compared with the SC group. For the SC group the median level of OPN in serum was 18.05 μg/L (range 5.54–35.15 μg/L).

### Correlation analysis

Spearman and Pearson correlation analyses for the biomarkers are presented without and with the Bonferroni correction in table 4. Biomarker variables that were correlated (r>20; see table 4) to gender or age were adjusted accordingly in the linear regression analysis.

**Table 4:** Spearman Correlation on categorical, nominal data and Pearson correlation on continuous data.

Characteristics	sCD163 serum	sCD163 CSF	sCD163 ratio	CXCL13 CSF	CXCL13 ratio	CXCL13 index	NEO CSF	NEO Ratio	NEO index	NfL CSF	OPN serum	OPN ratio	OPN index
**Spearman’s correlation coefficients, r, without and with the Bonferroni correction on ordinal variables**													
**RRMS & SC (n = 83)**	0.31	-0.23	-0.39	-0.58	-0.60	-0.56	-0.34	-0.43	-0.25	-0.65	-0.16	0.01	0.09
**Level of significance**	*/**-**	*/**-**	**/*	**/**	**/**	**/**	*/**-**	**/*	*/**-**	**/**	-/**-**	-/**-**	-/**-**
**PPMS & SC (n = 54)**	0.19	-0.44	-0.48	-0.32	-0.36	-0.31	-0.35	-0.43	-0.12	0.68	-0.40	0.20	0.25
**Level of significance**	-/**-**	**/**-**	**/*	*/**-**	*/**-**	*/**-**	*/**-**	*/**-**	-/**-**	**/**	*/**-**	-/**-**	-/**-**
**CIS & SC (n = 66)**	0.15	0.23	0.35	-0.46	-0.46	-0.46	-0.23	-0.33	-0.18	0.51	-0.50	0.29	0.28
**Level of significance**	-/**-**	-/**-**	*/**-**	**/*	**/*	**/*	-/**-**	*/**-**	-/**-**	**/*	**/*	*/**-**	*/**-**
**Gender (n = 125)**	-0.01	-0.11	-0.10	-0.02	-0.01	-0.03	-0.14	-0.11	0.10	-0.10	-0.30	0.25	0.30
**Level of significance**	-/**-**	-/**-**	-/**-**	-/**-**	-/**-**	-/**-**	-/**-**	-/**-**	-/**-**	-/**-**	*/**-**	*/**-**	**/**-**
**No. of attacks (n = 71)**	-0.17	-0.06	-0.01	0.31	0.36	-0.37	0.18	0.15	0.13	-0.32	-0.34	0.39	0.32
**Level of significance**	-/**-**	-/**-**	-/**-**	*/**-**	*/**-**	*/**-**	-/**-**	-/**-**	-/**-**	*/**-**	*/**-**	*/**-**	*/**-**
**Pearson’s correlation coefficients, r, without and with the Bonferroni correction on continuous variables**													
**Age (n = 125)**	0.21	0.23	-0.03	-0.22	-0.16	-0.21	-0.01	-0.13	-0.30	0.02	0.20	0.05	-0.08
**Level of significance**	*/**-**	*/**-**	-/**-**	*/**-**	-/**-**	*/**-**	-/**-**	-/**-**	**/**-**	-/**-**	*/**-**	-/**-**	-/**-**
**EDSS at time of diagnosis**	-0.16	0.26	0.32	0.16	0.17	0.14	0.23	0.24	0.03	0.42	0.22	-0.07	-0-15
**Level of significance**	-/**-**	*/**-**	**/*	-/**-**	-/**-**	-/**-**	*/**-**	*/**-**	-/**-**	**/**	*/*	-/*	-/*
**Disease duration (n = 86)**	-0.18	-0.08	0.10	-0.10	-0.08	-0.09	0.10	0.15	0.02	-0.06	-0.12	-0.00	-0.05
**Level of significance**	-/**-**	-/**-**	-/**-**	-/**-**	-/**-**	-/**-**	-/**-**	-/**-**	-/**-**	-/**-**	-/**-**	-/**-**	-/**-**
**Time since last attack (n = 71)**	0.10	0.00	-0.09	-0.32	-0.32	-0.32	-0.39	-0.23	-0.21	0.21	-0.04	-0.14	-0.15
**Level of significance**	-/**-**	-/**-**	-/**-**	*/**-**	*/**-**	*/**-**	**/**-**	-/**-**	-/**-**	-/**-**	-/**-**	-/**-**	**/**-**
**CSF Protein (n = 125)**	-0.07	0.44	0.35	0.18	0.18	0.04	0.22	0.26	-0.49	0.27	0.15	-0.01-	-0.41
**Level of significance**	-/**-**	**/**	**/*	*/**-**	*/**-**	-/**-**	*/**-**	*/**-**	**/**	*/**-**	-/**-**	-/**-**	**/**
**CSF Cells**	-0.20	0.29	0.37	0.50	0.48	0.46	0.51	0.52	0.30	0.37	-0.04	0.09	0.04
**Level of significance**	*/**-**	*/**-**	**/*	**/**	**/**	**/**	**/**	**/**	*/**-**	**/*	-/**-**	-/**-**	-/**-**
**IgG Index (n = 125)**	-0.22	0.23	0.34	0.5	0.51	0.54	0.41	0.40	0.43	0.38	0.01	0.03	0.10
**Level of significance**	*/**-**	*/**-**	**/*	**/**	**/**	**/**	**/**	**/**	**/**	**/*	-/**-**	-/**-**	-/**-**
**TNL (n = 125)**	-0.18	-0.01	0.16	-0.01	-0.00	-0.01	0.04	0.00	-0.05	0.02	-0.07	-0.08	-0.09
**Level of significance**	-/**-**	-/**-**	-/**-**	-/**-**	-/**-**	-/**-**	-/**-**	-/**-**	-/**-**	-/**-**	-/**-**	-/**-**	-/**-**

Table 4 shows the results of the Spearman Correlation on categorical, nominal data and Pearson correlation on continuous data. Variables that are ordinal were analyzed with Spearman correlation without and with the Bonferroni correction and continuous variables were analyzed using Pearson correlation without and with the Bonferroni correction. In the top row only biomarkers that were significantly correlated to patient groups have been included. Thus, each biomarker value was first correlated to the three groups: RRMS & SC, PPMS & SC, and CIS & SC, and then further correlated to all the demographic parameters. The correlation coefficient, *r*, is displayed with the significance levels indicated both without / and with the Bonferroni correction by * for p<0.05, and ** for p<0.001. All p-values are presented in tables B-Y in S1 Dataset. Abbreviations: RRMS (relapsing-remitting MS), PPMS (primary-progressive MS), CIS (clinically isolated syndrome), SC (symptomatic controls), n (number of persons), CSF (cerebrospinal fluid), NfL (neurofilament light polypeptide), TNL (total number of MRI white matter lesions), NEO (neopterin), OPN (osteopontin).

Patients in the PPMS group were significantly older than patients in the RRMS, CIS and SC groups; and levels of sCD163 in both serum and CSF were found to correlate with age, however this was not the case for the sCD163 ratio.

The levels of sCD163 in serum and CSF were not significantly correlated to gender or any of the clinical measures: time since last attack, EDSS, mean no. of relapses, mean disease duration, enhancing lesions or the total number of MRI white matter lesions (TNL). There were moderate to strong correlations between most biomarker variables and CSF protein, CSF cells and IgG index.

Pearson pairwise correlation analysis of biomarkers is presented without and with the Bonferroni correction in table 5. sCD163 CSF and the sCD163 ratio were correlated to most of the other biomarkers, moderately correlated to OPN CSF, NEO CSF and the NEO ratio (p<0.001). Interestingly, NEO CSF and the NEO ratio were moderately correlated to CXCL13 CSF and the CXCL13 ratio. NfL CSF was weakly correlated to sCD163, CXCL13 and NEO levels. NfL CSF and index was also moderately correlated to EDSS.

**Table 5:** Pearson pairwise correlation analysis without and with the Bonferroni correction of biomarkers: sCD163, CXCL13, NEO, NfL, and OPN.

Characteristics	sCD163 serum	sCD163 CSF	sCD163 ratio	CXCL 13 serum	CXCL13 CSF	CXCL13 ratio	NEO serum	NEO CSF	NEO Ratio	NfL CSF	OPN serum	OPN CSF	OPN ratio
**sCD163 serum**	1.00												
**sCD163 CSF**	0.08	1.00											
**Level of significance**	-/-												
**sCD163 ratio**	-0.61	0.57	1.00										
**Level of significance**	**/**	**/**											
**CXCL 13 serum**	-0.14	-0.05	0.15	1.00									
**Level of significance**	-/-	-/-	-/-										
**CXCL13 CSF**	-0.10	0.22	0.22	0.06	1.00								
**Level of significance**	-/	*/-	*/-	-/									
**CXCL13 ratio**	-0.08	0.20	0.18	-0.04	0.98	1.00							
**Level of significance**	-/-	*/-	*/-	-/-	**/**								
**NEO serum**	0.20	0.04	-0.13	-0.08	0.07	0.08	1.00						
**Level of significance**	*/-	-/-	-/-	-/-	-/-	-/-							
**NEO CSF**	-0.09	0.31	0.29	0.02	0.57	0.54	0.35	1.00					
**Level of significance**	-/-	**/*	*/-	-/-	**/-	**/**	**/*						
**NEO Ratio**	-0.24	0.30	0.40	0.08	0.50	0.45	0.30	0.74	1.00				
**Level of significance**	*/-	**/-	**/**	-/-	**/**	**/**	**/-	**/**					
**NfL CSF**	-0.16	0.21	0.26	0.02	0.26	0.20	-0.00	0.22	0.24	1.00			
**Level of significance**	-/-	*/-	*/-	-/-	*/-	*/-	-/-	*/-	*/-				
**OPN serum**	-0.00	0.25	0.25	-0.09	0.02	0.01	-0.03	0.04	0.04	0.25	1.00		
**Level of significance**	-/-	*/-	*/-	-/-	-/-	-/-	-/-	-/-	-/-	*/-			
**OPN CSF**	0.08	0.42	0.22	-0.05	0.35	0.34	0.22	0.40	0.21	0.36	0.10	1.00	
**Level of significance**	-/-	**/**	*/-	-/-	**/*	**/*	*/-	**/**	*/-	**/*	-/-		
**OPN ratio**	0.05	0.22	0.09	-0.01	0.19	0.19	0.31	0.28	0.10	0.11	-0.47	0.72	1.00
**Level of significance**	-/-	*/-	-/-	-/-	*/-	*/-	**/*	*/-	-/-	-/-	**/**	**/**	

Table 5 shows the results from the Pearson pairwise correlation analyses without and with the Bonferroni correction of biomarkers and their intercorrelation. The correlation coefficient, r, is displayed with the significance levels without / and with the Bonferroni corrections marked by * for p<0.05, and ** for p<0.001. All p-values are presented in tables Z and AA in S1 Dataset. Abbreviations: CSF (cerebrospinal fluid), NfL (neurofilament light polypeptide), NEO (neopterin), OPN (osteopontin).

### Linear regression with relevant age and/or gender correction

Results from the linear regression analysis are presented in table 6 (see table AB in S1 Dataset for output). Biomarker variables with high group correlations (r >0.4; p<0.001) are shown.

The geometric mean (GM) values of the sCD163 ratio was significantly increased for the patients with RRMS: 1.36 fold (95%CI: 1.17–1.59; p<.001), CIS: 1.33 fold (95%CI: 1.07, 1.65; p = 0.010), and especially PPMS: 1.57 fold (95%CI: 1.23–1.97; p<0.001).

**Table 6:** Results from linear regression analyses of differences in levels of biomarkers between patients with MS/CIS and SC.

Characteristics	RRMS	PPMS	CIS
**No. of Subjects (total = 125)**	n = 44	n = 15	n = 27
**CSF sCD163, GM fold increase compared with SC (95% CI)**	1.10 (1.00.– 1.21)	1.23 (1.06–1.44)	1.18 (1.03–1.35)
**(p-values)**	(0.05)	(0.008)*	(0.015) *
**sCD163 ratio, GM fold increase compared with SC (95% CI)**	1.36 (1.17–1.59)	1.57 (1.23–1.97)	1.33 (1.07–1.65)
**(p-values)**	(<0.001)*	(<0.001)*	(0.010)*
**CSF CXCL13, GM fold increase compared with SC (95% CI)**	12.84 (4.13–39.88)	5.18 (1.25–21.41)	7.33(2.19–24.52)
**(p-values)**	(<0.001)*	(0.023)*	(0.001)*
**CXCL13 ratio, GM fold increase compared with SC (95% CI)**	13.04 (4.19–40.58)	5.52 (1.29–23.53)	6.86 (1.94–24.26)
**(p-values)**	(<0.001) *	(0.021)*	(0.003)*
**CXCL13 index, GM fold increase compared with SC (95% CI)**	11.30 (3.62–35.23)	4.65 (1.02–21.13)	6.08 (1.80–20.51)
**(p-values)**	(<0.001)*	(0.047)*	(0.004)*
**NEO ratio, GM fold increase compared with SC (95% CI)**	1.44 (1.22–1.70)	1.35 (1.14–1.60)	1.28 (1.08–1.54)
**(p-values)**	(<0.001)*	(<0.001)*	(0.006)*
**CSF NfL, GM fold increase compared with SC (95% CI)**	2.52 (1.95–3.26)	2.49 (1.96–3.18)	2.18 (1.55–3.09)
**(p-values)**	(<0.001)*	(<0.001)*	(<0.001)*
**Serum OPN, GM fold increase compared with SC (95% CI)**	1.20 (0.98–1.47)	1.20 (0.93–1.55)	1.62 (1.32–1.98)
**(p-values)**	(0.073)	(0.16)	(<0.001)*

Table 6 shows the results of linear regression analyses on parameters identified in the previous correlation analysis. The regression analyses were performed on log transformed data and if the parameter was correlated to age and/or gender in the correlation analysis these were included in the linear regression. Fold differences in geometric means (GM) and CIs are shown for RRMS, PPMS, and CIS compared with the SC group. The level of significance is shown by the p-value and * marks a significant difference between groups. Abbreviations: RRMS (relapsing-remitting MS), PPMS (primary-progressive MS), CIS (clinically isolated syndrome), SC (symptomatic controls), n (number of persons), CSF (cerebrospinal fluid), CI (confidence interval) NfL (neurofilament light polypeptide), NEO (neopterin), OPN (osteopontin). The data on sCD163 have previously been presented in [18].

Of the other variables, most notably the CXCL13 ratio was elevated 13 fold (95%CI: 4.19–39.88, p<0.001) in patients with RRMS, but also significantly elevated in patients with PPMS: 5.52 fold (95%CI: 1.29–23.53; p = 0.021) and CIS: 6.86 fold (95%CI: p = 0.003). The NEO ratio was significantly elevated in all groups, especially RRMS: 1.44 (95%CI: 1.22–1.70; p<0.001). The NfL CSF was notably increased for patients with PPMS: 2.49 fold (95%CI: 1.96–3.18; p<0.001).

It is noteworthy, that OPN was a variable in which serum levels were elevated compared with the SC group, significantly for the CIS group: 1.62 fold (95%CI: 1.32–1.98; p<0.001).

**Fig 3:**
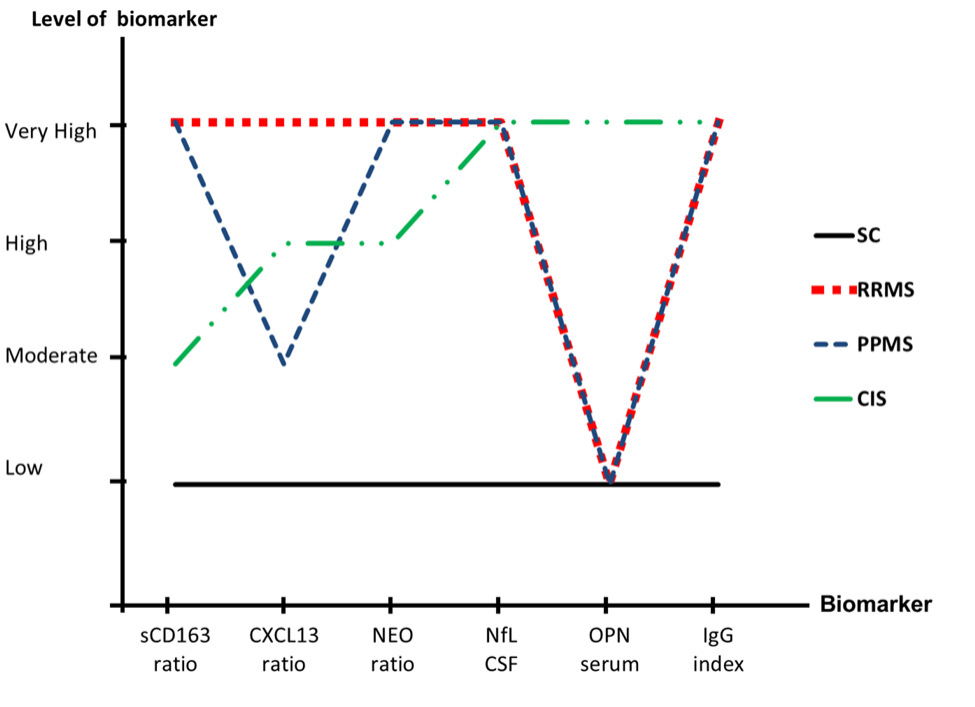
Fig 3 shows the biomarker profile for each group of patients. The biomarker profile was constructed as an overview representing the major characteristics of each group in the cohort. The profile was determined by categorizing the GM levels of the sCD163 ratio, the CXCL13 ratio, the NEO ratio, the NfL CSF, the OPN serum, and the IgG index for each patient group in the following manner: Very high (significantly increased GM, p <0.001); High (significantly increased GM, p <0.01); Moderate (significantly increased GM, p <0.05); Low (zero is included in the CI). Abbreviations: NEO (neopterin), NfL (neurofilament light polypeptide), OPN (osteopontin), SC (symptomatic controls), RRMS (relapsing-remitting MS), PPMS (primary-progressive MS), CIS (clinically isolated syndrome), CI (confidence interval), GM (geometric means).

**Fig 4:**
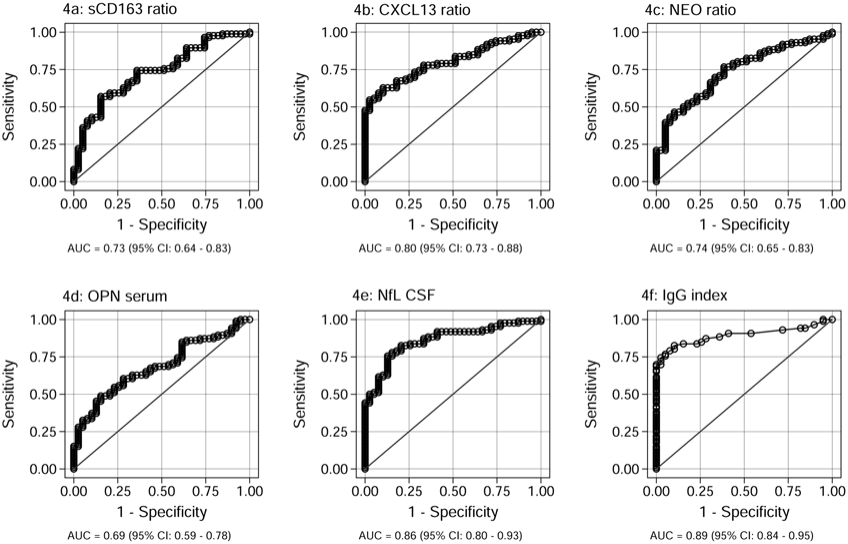
ROC curves for the sCD163 ratio, the CXCL13 ratio, the NEO ratio, the NfL CSF, the OPN serum, and the IgG index. Fig 4 shows the ROC curves generated for the sCD163 ratio (4a), the CXCL13 ratio (4b), the NEO ratio (4c), the NfL CSF (4d), the OPN serum (4e), and the IgG index (4f). The data on sCD163 have previously been presented in [18]. AUC, with 95% CI, is given for each parameter. The parameter results for patients with RRMS, PPMS, and CIS are combined as true positives and plotted against SC as true negatives. The diagonal dividing the ROC space represents the random event. Abbreviations: ROC (receiver operating characteristic), AUC (area under the curve), RRMS (relapsing-remitting MS), PPMS (primary-progressive MS), CIS (clinically isolated syndrome), SC (symptomatic controls), CSF (cerebrospinal fluid), CI (confidence interval).

**Fig 5:**
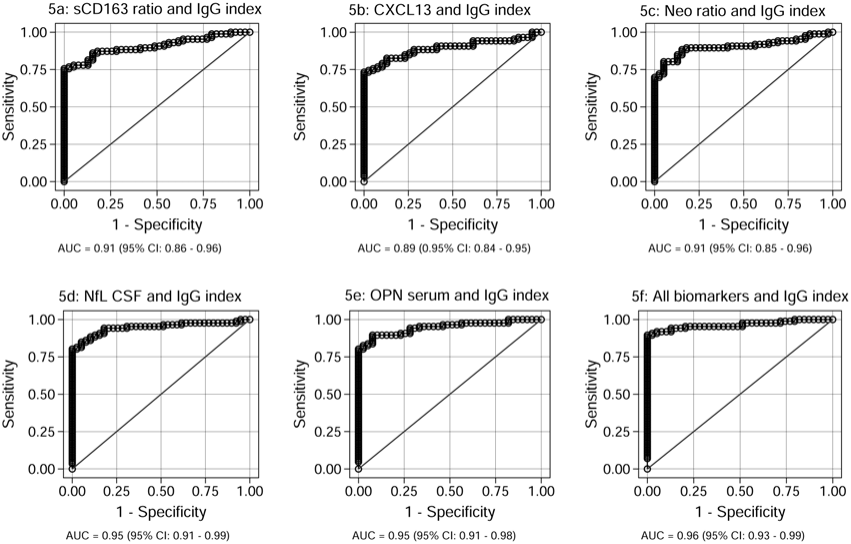
ROC curves for the sCD163 ratio, the CXCL13 ratio, the NEO ratio, the NfL index, and the OPN serum combined with the IgG index individually as well as a combined ROC curve. Fig 5 shows the combined ROC curves generated for the IgG index and the sCD163 ratio (5a), the CXCL13 ratio (5b), the NEO ratio (5c), the NfL CSF (5d), the OPN serum (5e), and All biomarkers (5f). AUC, with 95% CI, is given for each parameter. The parameter results for patients with RRMS, PPMS, and CIS are combined as true positives and plotted against SC as true negatives. The diagonal dividing the ROC space represents the random event. Abbreviations: ROC (receiver operating characteristic), AUC (area under the curve), RRMS (relapsing-remitting MS), PPMS (primary-progressive MS), CIS (clinically isolated syndrome), SC (symptomatic controls), CI (confidence interval).

The biomarkers with the optimal differentiating properties were established from the regression analysis: the sCD163 ratio, the CXCL13 ratio, the NEO ratio, the NfL CSF, and the OPN serum. These parameters were further analyzed in a biomarker profile (Fig 3), a ROC analysis (Fig 4), and using logistic regression analyses (Fig 5).

### Biomarker profile

The biomarker profile was determined by categorizing the GM for each patient group in the following manner: Very high (significantly increased GM, p <0.001); High (significantly increased GM, p = [0.01;0.001]; Moderate (significantly increased GM, p =] 0.05; 0.01]; and Low (zero is included in the CI). Fig 3 demonstrates a distinct profile for each patient group, distinguishing all patient groups from the SC group and also, to some extent, differentiating between patients with RRMS, PPMS, and CIS.

### ROC analysis and logistic regression on biomarker performance

To examine the sensitivity and specificity of each biomarker a ROC analysis was performed for each biomarker (Fig 4). The sCD163 ratio (Fig 4a) had an area under the curve (AUC) of 0.73 (95%CI: 0.64–0.83) and this was higher than for OPN serum (Fig 4d; AUC 0.69 (95%CI: 0.59–0.78)), equal to the NEO ratio (Fig 4c; AUC 0.74 (95%CI: 0.65–0.83)) and lower than the CXCL13 ratio (Fig 4b; 0.80 (95%CI: 0.73–0.88)), and the NfL CSF (Fig 4e; AUC 0.86 (95%CI: 0.80–0.93)). All individual biomarkers had a lower performance than the IgG index (Fig 4f; AUC 0.89 (95%CI: 0.84–0.95)).

The ROC analyses were followed by a logistic regression model (see methods and table AD in S1 Dataset) in which each biomarker was combined with the IgG index, and finally in a combined analysis with all biomarkers (Fig 5). The combined analyses of sCD163 and the IgG index (Fig 5a) gave an AUC of 0.91 (95%CI: 0.86–0.96) which was higher than the CXCL13 levels and the IgG index combined (Fig 5b; AUC 0.89 (95%CI: 0.84–0.95)), equal to the NEO ratio (Fig 5c; AUC: 0.91 (95%CI: 0.85–0.96), and lower than the NFL index (Fig 5d; AUC: 0.95 (95%CI: 0.93–0.99)) and the OPN serum levels (Fig 5e; AUC: 0.95 (95%CI: 0.91–0.98). The combined biomarker panel (Fig 5f) had an AUC of 0.97 (95%CI: 0.93–0.99)—higher than that of all the individual biomarker AUCs and also higher than all of the individual biomarkers combined with the IgG index alone.

## Discussion

The biomarkers selected for this study have all documented discriminating potency providing differentiation between diseased and healthy subjects. As pointed out by Xia *et al* [61] this sort of study primarily focuses on the development of a biomarker panel. As a marker of inflammation, sCD163 levels are elevated in a range of diseases where inflammation contributes to the morbidity and disability [62, 63]. As a marker for monocyte / macrophage activity in disease sCD163 has proven to be a reliable biomarker [64]. In our recently published study [18], we demonstrated that sCD163 is a fair marker for MS disease, and so, the contribution of this study to MS diagnostics is not the individual biomarker levels as such, but rather the increased discriminatory powers in the combination of biomarkers.

### Contributions of sCD163 to a panel of biomarkers for inflammation and axonal degeneration/damage

For sCD163 the results constitute a post-hoc analysis of already published data [18]. In the present study we show that sCD163 contributes to the differentiation between SC and patients diagnosed with CIS or MS as shown in Fig 3. The ROC analyses of the individual biomarkers shows that with respect to specificity and sensitivity, the sCD163 ratio scores on a par with the established biomarkers regarding diagnostic properties (see Fig 4a–4f). According to the criteria outlined by Xia *et al* [61] none of the biomarkers analysed could be categorized as excellent (AUC = 0.9–1.0); the IgG index, the NfL index, and the CXCL13 ratio could be categorized as good (AUC = 0.8–0.9), the sCD163 ratio and the NEO ratio could be categorized as fair (AUC = 0.7–0.8), whereas, finally, the OPN serum level could be categorized as poor (AUC = 0.6–0.7) [61]. However, as demonstrated in Fig 5a–5e, the panel of combined biomarkers performs the diagnostic differentiation to a very high level, elevating the diagnostic properties to excellent for most biomarkers (only the CXCL13 ratio is exempted), when combined with the IgG index. The combination of all tested biomarkers (Fig 5f) resulted in an AUC of 0.97, illustrating that in a multifactorial disease, such as MS, the combination of multiple biomarkers into a singularised multivariable model provides a high level of diagnostic discrimination and reliability. Interestingly, in the present study OPN serum shows a poor discriminatory capacity when viewed as a single marker, yet combined with IgG index it becomes excellent. This is probably due to the fact that it OPN is especially elevated in serum of patients with CIS (Fig 2n) as is not the case for the IgG index.

Overall, we find it noteworthy that (except for OPN) the biomarker ratios and indexes (i.e. levels in CSF in relation to levels in serum) were optimal in differentiating between patient groups rather than levels in either CSF or serum. The well-established MS diagnostic marker, the IgG index, also measures a relation between levels in the CNS and in the systemic circulation. This underscores that measuring biomarker levels concomitantly in both compartments is advantageous.

In the present study patients with PPMS had significantly elevated sCD163 CSF median levels compared with SC. This is also the case for the NEO CSF levels. Interestingly, the levels of sCD163 and NEO in serum and CSF are decreased / increased in similar ways (Fig 2a, 2b, 2i and 2j). It has recently been reported, that the release of NEO triggers the release of TNFα, which is associated with the release of sCD163 [21]. The levels of NEO and sCD163 in both serum and CSF were found to be comparable, supporting a concerted inflammatory response. Especially in PPMS, where sCD163 is known to be up regulated during the pro-inflammatory response [65], and the release of NEO is known to contribute to the orchestration of oxidative stress [37].

For the other biomarkers analysed, the results were as follows: the median levels of the CXCL13 ratio were significantly increased for all patient groups compared with SC. This increase attained the highest level of significance for patients with RRMS, followed by CIS and PPMS. This is in agreement with the general hypothesis that the pathological mechanism in these two groups is mainly driven by inflammation of which CXCL13 is a marker [66].

There were no significant differences between GM levels of NfL in patients with PPMS, CIS and RRMS, yet they were all significantly elevated compared with SC. This is interesting, as NfL has been proposed as a marker for relapse in MS [67]. However, that study had some overlap in NfL levels between groups as is also the case in the present study. The levels of NfL were also significantly correlated to *no*. *of attacks* (r = 0.32) but there was no correlation to *time since last attack* (table 4).

For OPN, we found that patients with CIS had significantly elevated GMs of OPN in serum as compared with SC, as was also the case in the age adjusted linear regression model (table 6). Furthermore, the GM levels in CIS were also significantly elevated when compared with the GM levels in the RRMS and PPMS groups. It is noteworthy, that in a recent study by Kivisakk et al [54] the CIS patients had the lowest levels of OPN compared with patients with MS. However, that study is based on a patient cohort undergoing immune-modulating treatment, and thus the two cohorts are not comparable. The present study substantiates that PPMS also has an inflammatory component, as illustrated by the significantly elevated levels of the CXCL13 ratio, the sCD163 ratio and the NEO ratio as compared with the SC groups. The role of macrophages/microglia in progressive MS disease has recently been reviewed [68] and it is suggested that the damage unfolded during progression of the MS disease is due both to chronic inflammation and to neurodegeneration [69]. Axonal transection is a common feature in MS pathology [70] and axonal damage/loss plays a key role in MS disability development. Thus, macrophages/microglia are intimately related to axonal damage in MS lesions and as MHC class II-positive cells [71] they are abundantly present in acute and chronically active MS lesions [70, 72]. It has been proposed that macrophages/microglia are involved in T cell activation [73] but also that they actively hinder lymphocyte proliferation illustrating the complex orchestration of pathology where macrophages/microglia play a pivotal role.

We are aware, that the results obtained with the present panel of biomarkers needs to be validated in a larger, independent cohort before application into a clinical setting; particularly as the sample numbers in the present patient groups are rather small for PPMS and SPMS. Extrapolation of results of the analyses to a wider range of patients including patients with i.e. non-demyelinating neurological or infectious disease of the CNS should also be explored in further studies.

In conclusion we propose that the sCD163 ratio contributes as a biomarker in differentiating newly diagnosed patients with MS/CIS from SC. The biomarker panel showed different profiles for each patient group and could be a valuable tool for clinical differentiation of MS subgroups. All tested biomarkers in the present study were significantly correlated to RRMS, PPMS, and/or CIS and their combined ROC curve analysis demonstrated excellent diagnostic discriminatory power. Finally, the study supports that PPMS has an inflammatory component as the levels of all biomarkers (except OPN) were significantly elevated in PPMS.

## Supporting Information

S1 Dataset Comprise Tables A—AD. (DOCX)

S1 Previous Publication Reference [18] by Stilund *et al*. (PDF)
