# Supplementary material for: Biomarkers of Inflammation and Axonal Degeneration/Damage in Patients with Newly Diagnosed Multiple Sclerosis: Contributions of the Soluble CD163 CSF/Serum Ratio to a Biomarker Panel
Source: PLoS One. 2015 Apr 10;10(4):e0119681. doi: 10.1371/journal.pone.0119681 (PMC4393241; doi:10.1371/journal.pone.0119681)
Supplement: S1 Dataset — (DOCX) [file pone.0119681.s001.docx]

**S1_Dataset**

**Table A. Basic Data.**

**Table A. Basic Data.** Excel file above (double click to activate) contains all basic data for this paper. Abbreviations: RRMS (relapsing-remitting MS), PPMS (primary-progressive MS), SPMS (secondary-progressive MS), CIS (clinically isolated syndrome), SC (symptomatic controls with normal or abnormal MRI), n (number of persons), CSF (cerebrospinal fluid), y (years), d (days), Gender (1=male; 2=female) TNL (Total number of white matter lesions).

**Do-file and output for correlation analysis in STATA.**

**Table B. Do-file and output of the Spearman correlation analysis without the Bonferroni correction on RRMS and SC.**

**
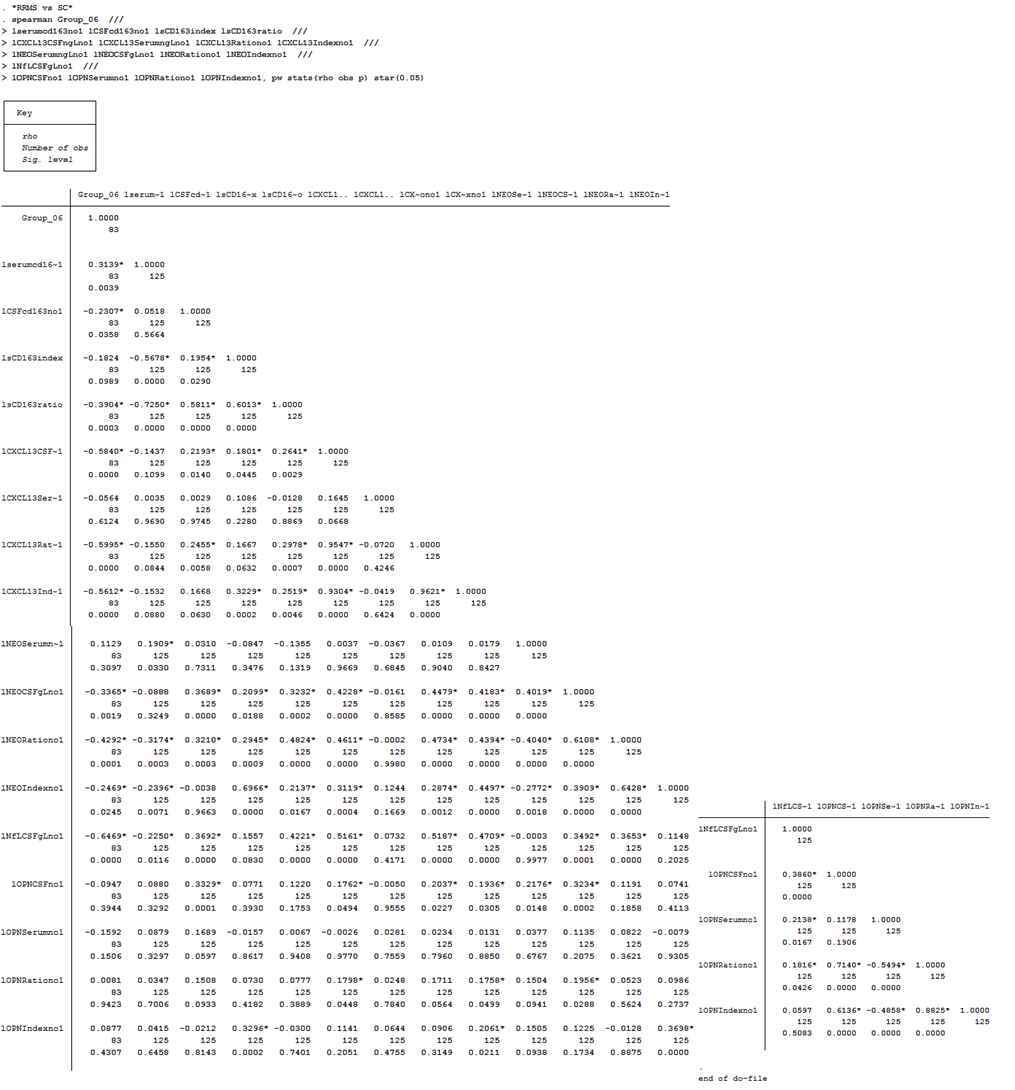
**

**Table C. Do-file and output of the Spearman correlation analysis with the Bonferroni correction on RRMS and SC.**

**
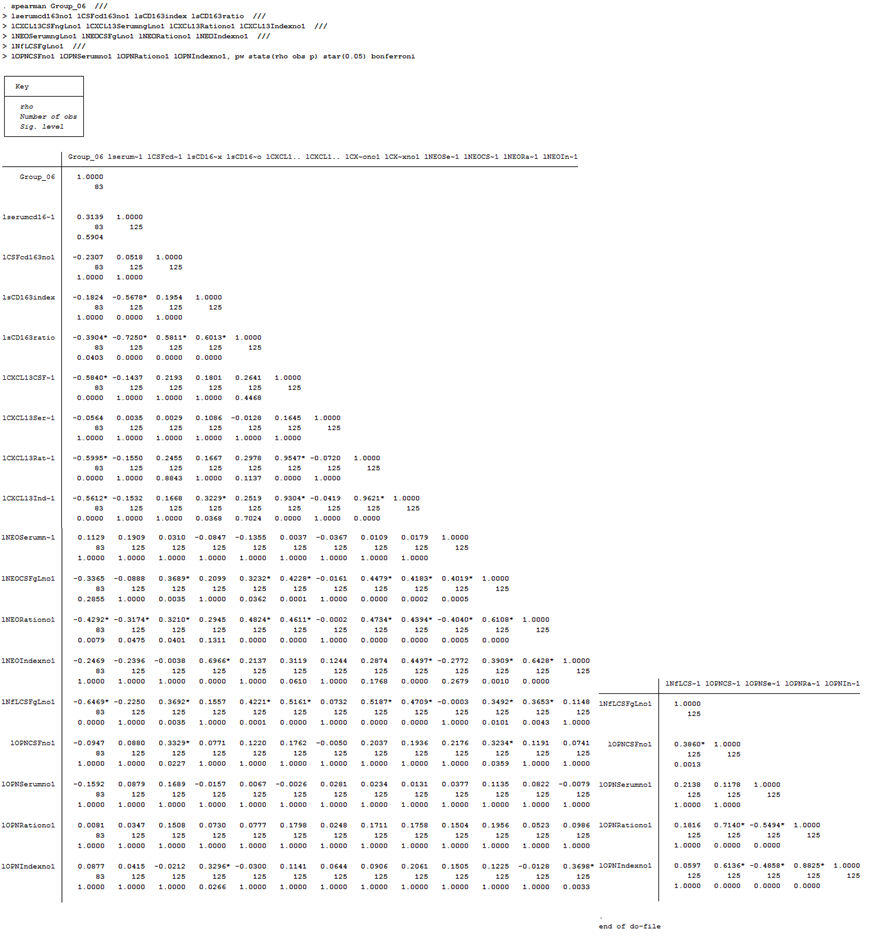
**

**Table D. Do-file and output of the Spearman correlation analysis without the Bonferroni correction on PPMS and SC.**

**
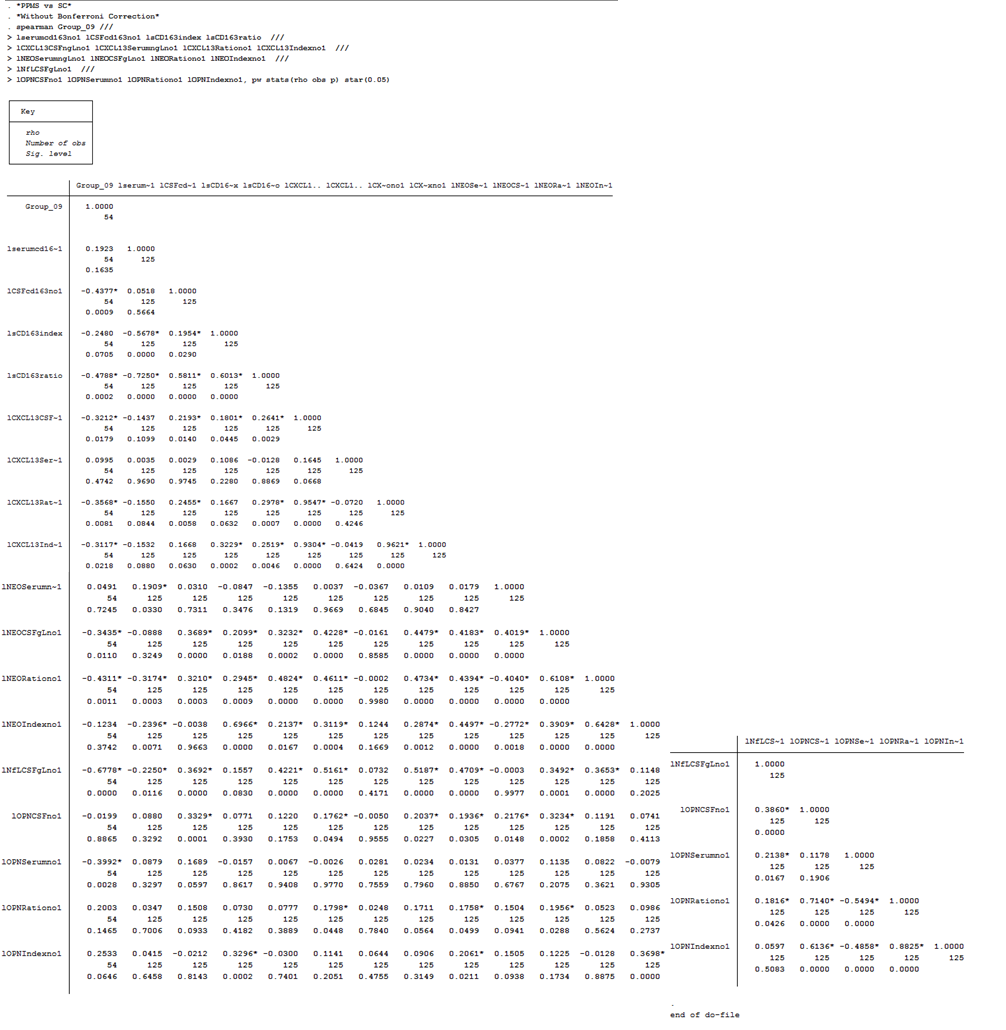
**

**Table E. Do-file and output of the Spearman correlation analysis with the Bonferroni correction on PPMS and SC.**

**
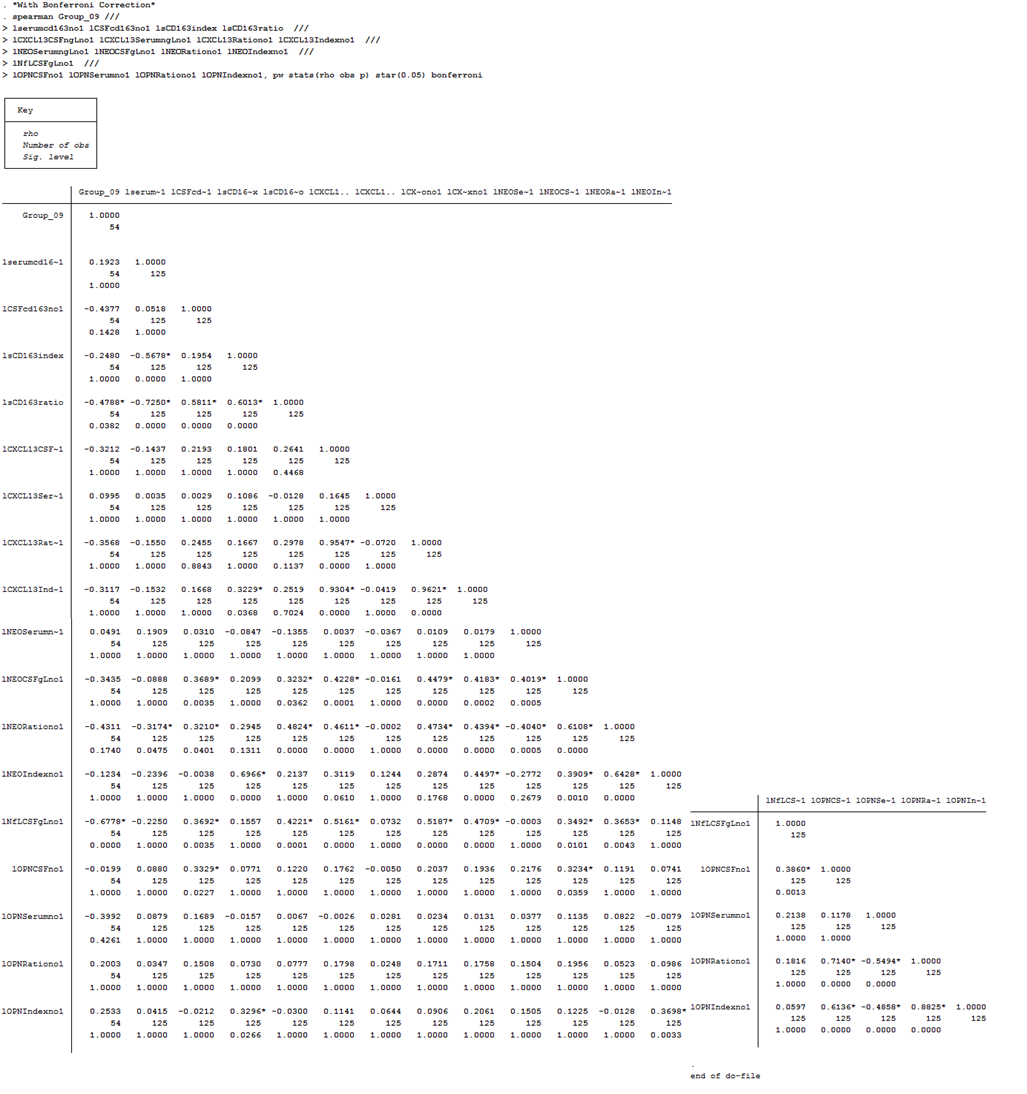
**

**Table F. Do-file and output of the Spearman correlation analysis without the Bonferroni correction on CIS and SC.**

**
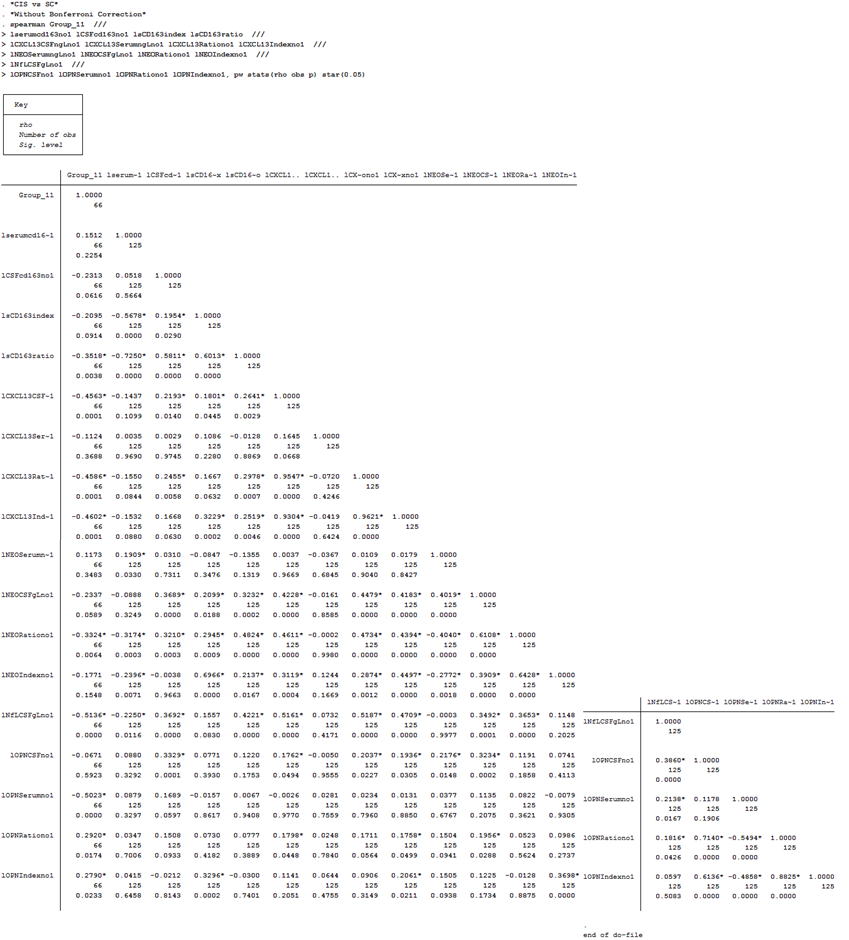
**

**Table G. Do-file and output of the Spearman correlation analysis with the Bonferroni correction on CIS and SC.**

**
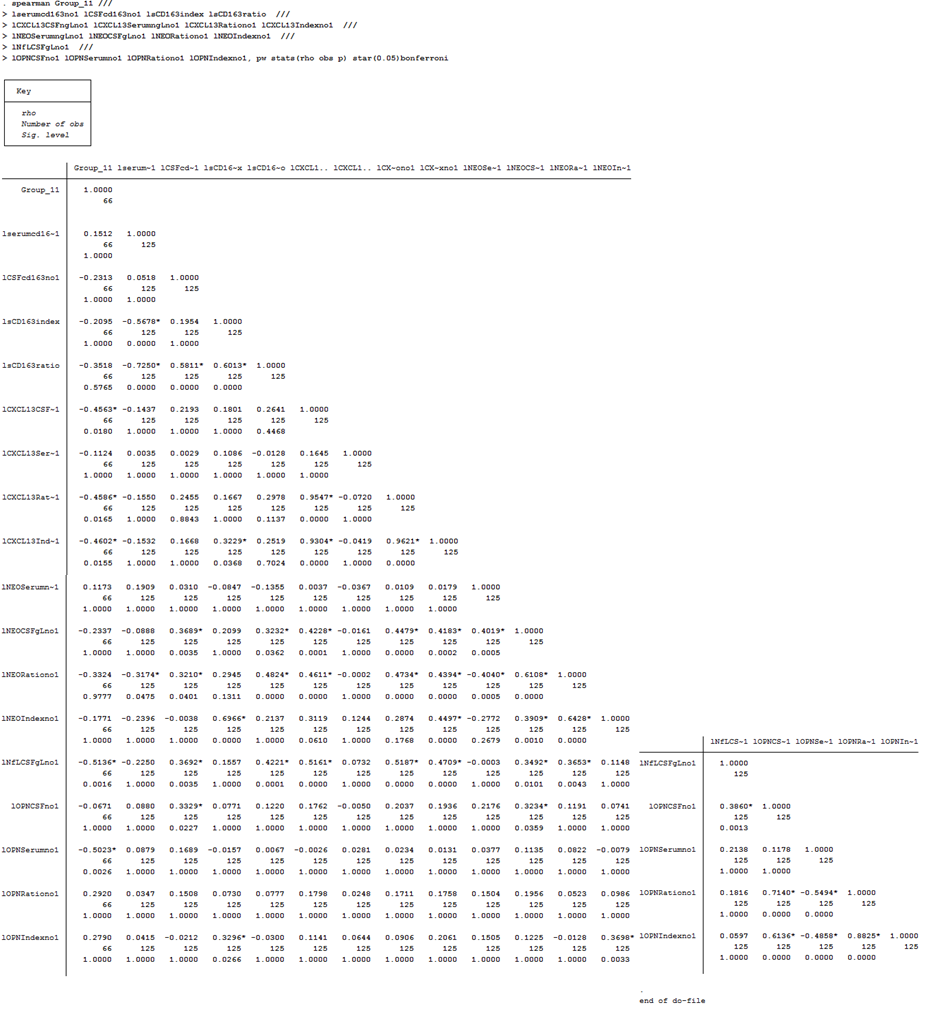
**

**Table H. Do-file and output of the Spearman correlation analysis without the Bonferroni correction on Gender and Number of Attacks.**

**
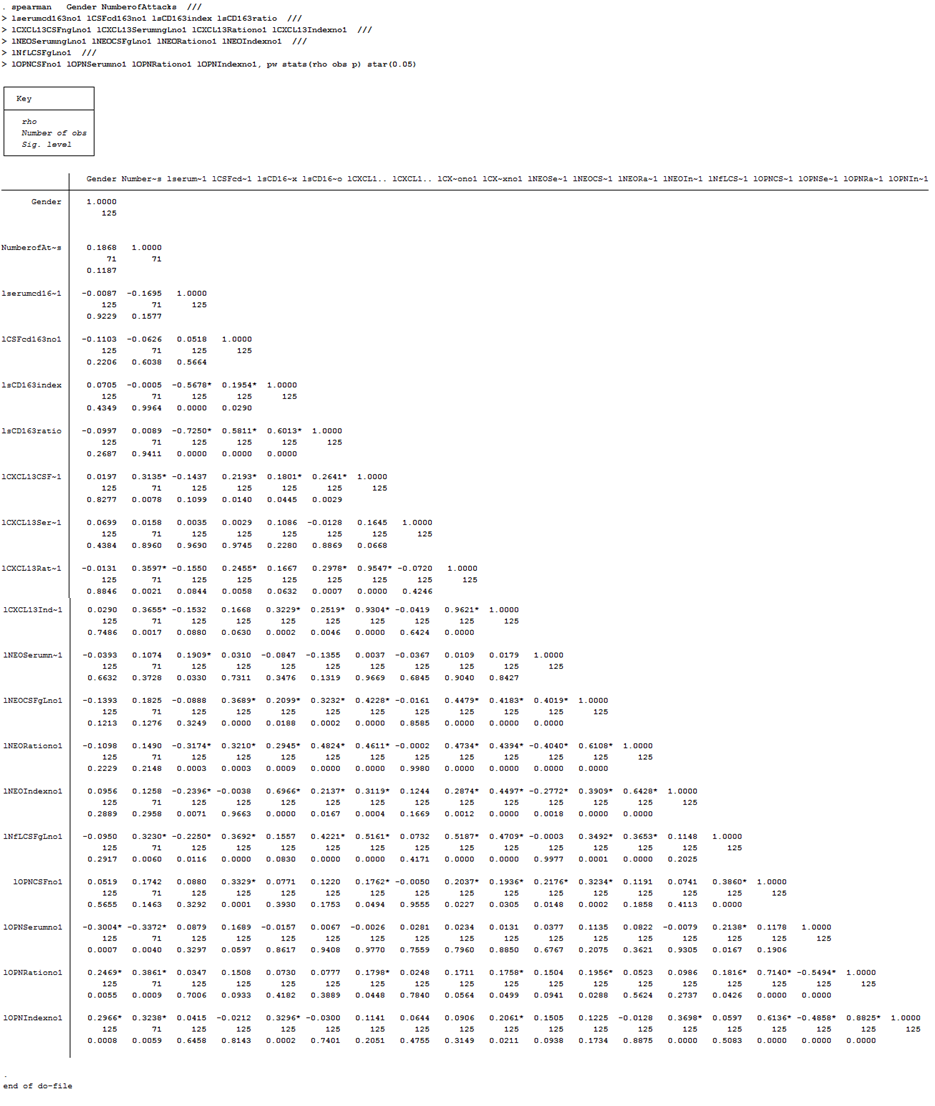
**

**Table I. Do-file and output of the Spearman correlation analysis with the Bonferroni correction on Gender and Number of Attacks.**

**
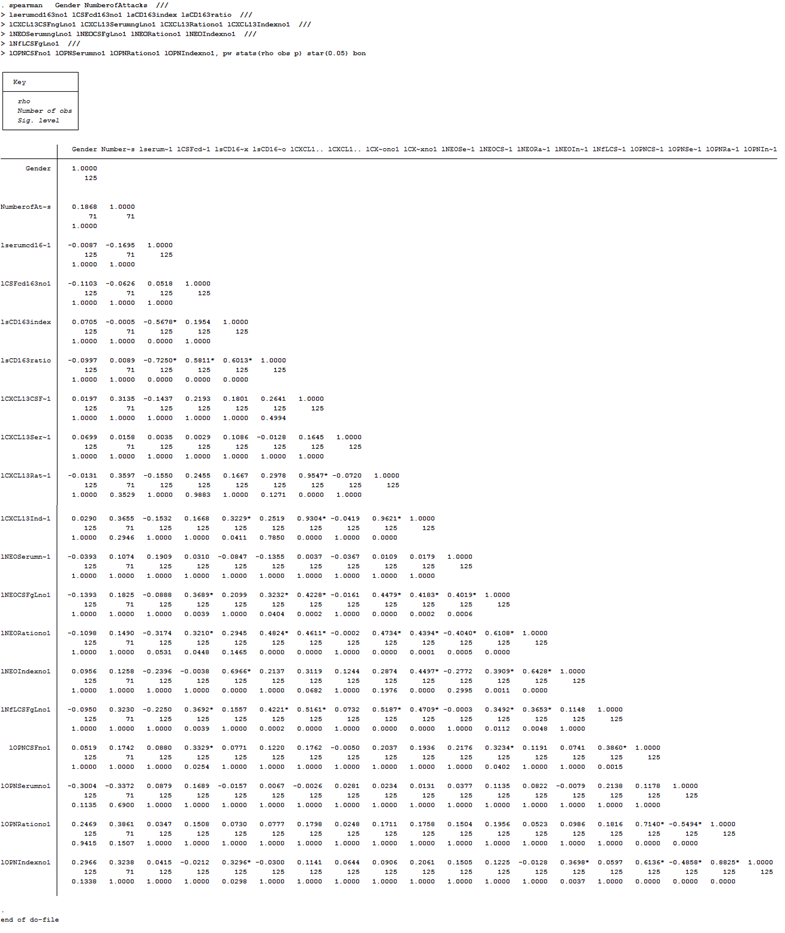
**

**Table J. Do-file and output of the Pearson correlation analysis without the Bonferroni correction on Age.**

**
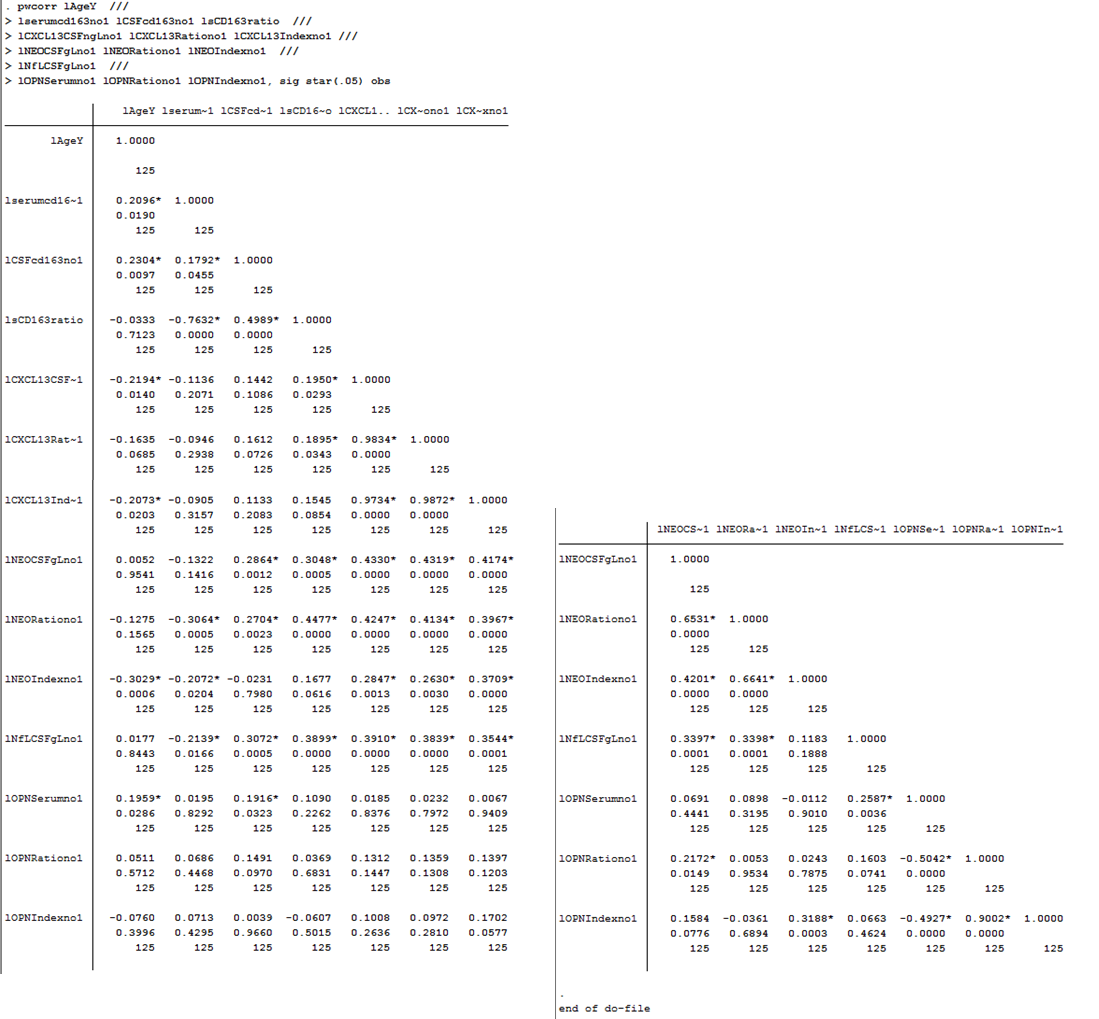
**

**Table K. Do-file and output of the Pearson correlation analysis with the Bonferroni correction on Age.**

**
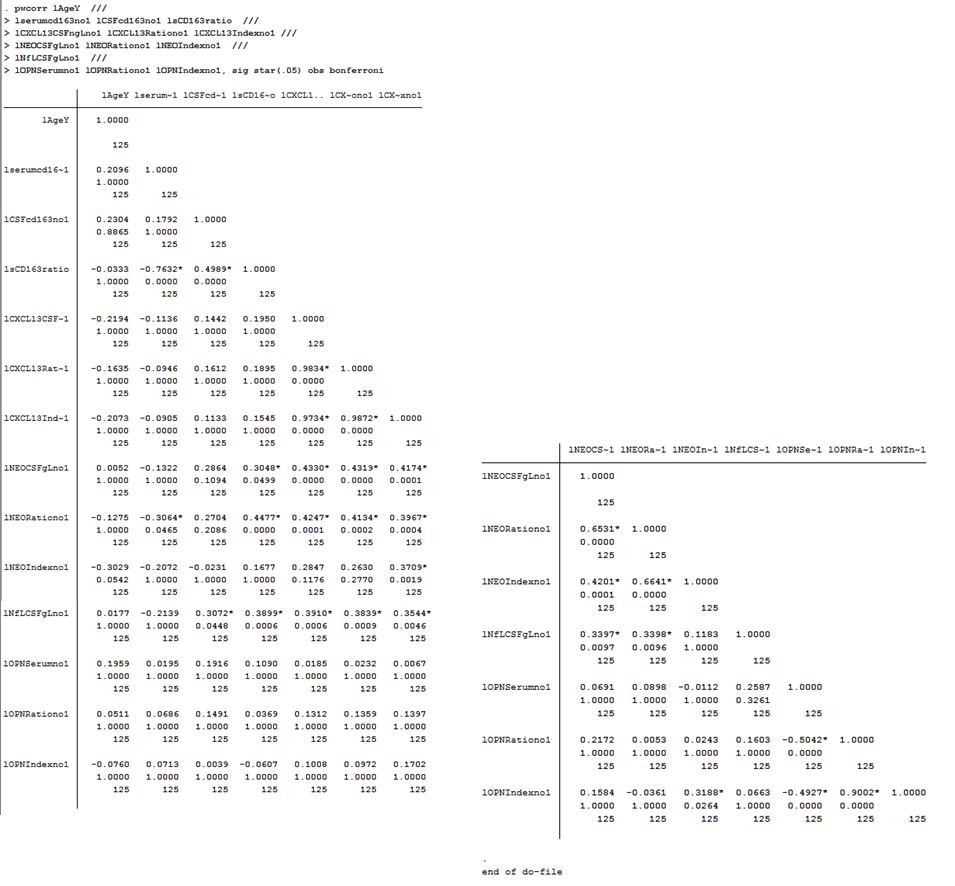
**

**Table L. Do-file and output of the Pearson correlation analysis without the Bonferroni correction on EDSS at time of diagnosis.**

**
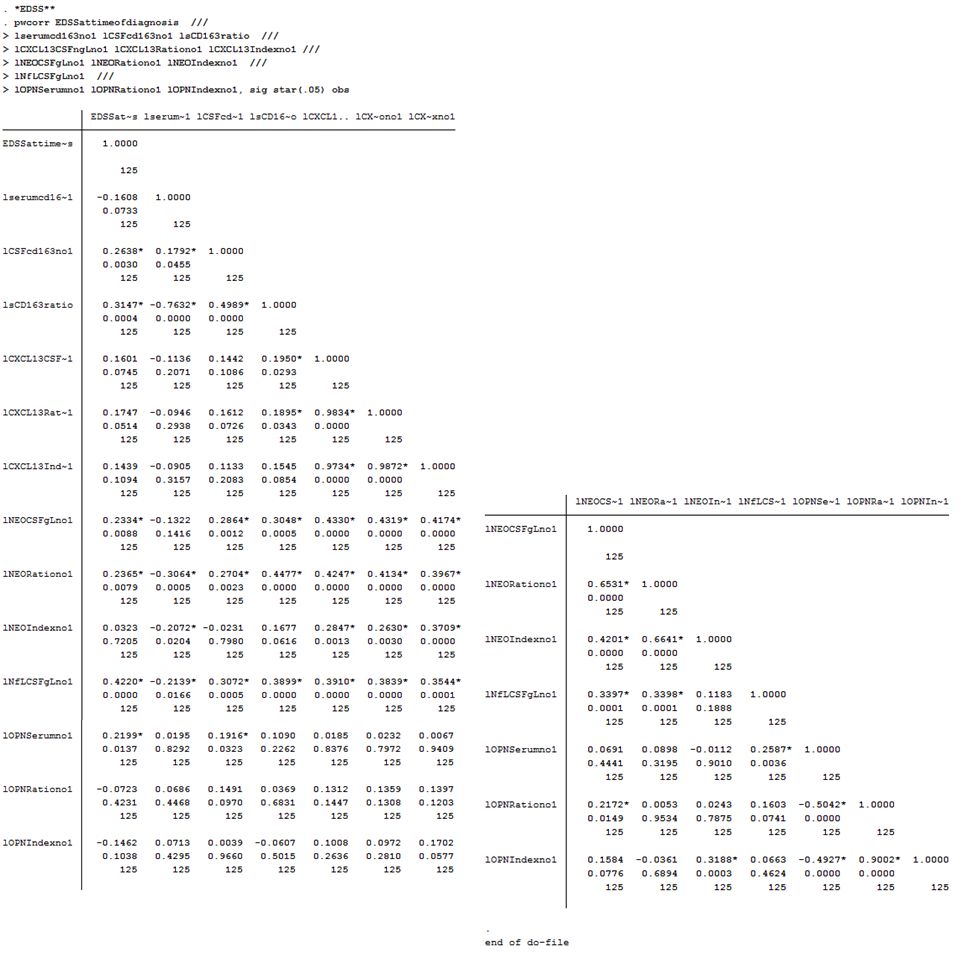
**

**Table M. Do-file and output of the Pearson correlation analysis with the Bonferroni correction on EDSS at time of diagnosis.**

**
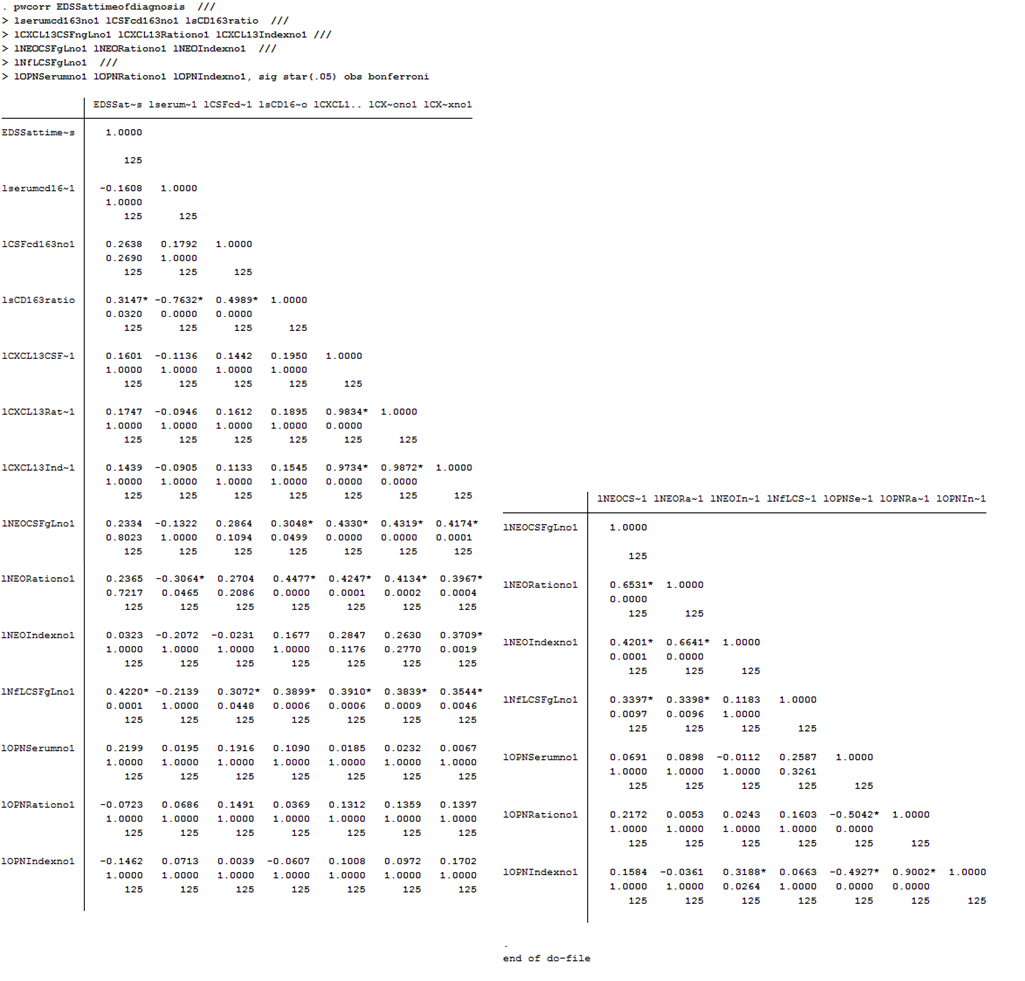
**

**Table N. Do-file and output of the Pearson correlation analysis without the Bonferroni correction on Disease duration (months).**

**
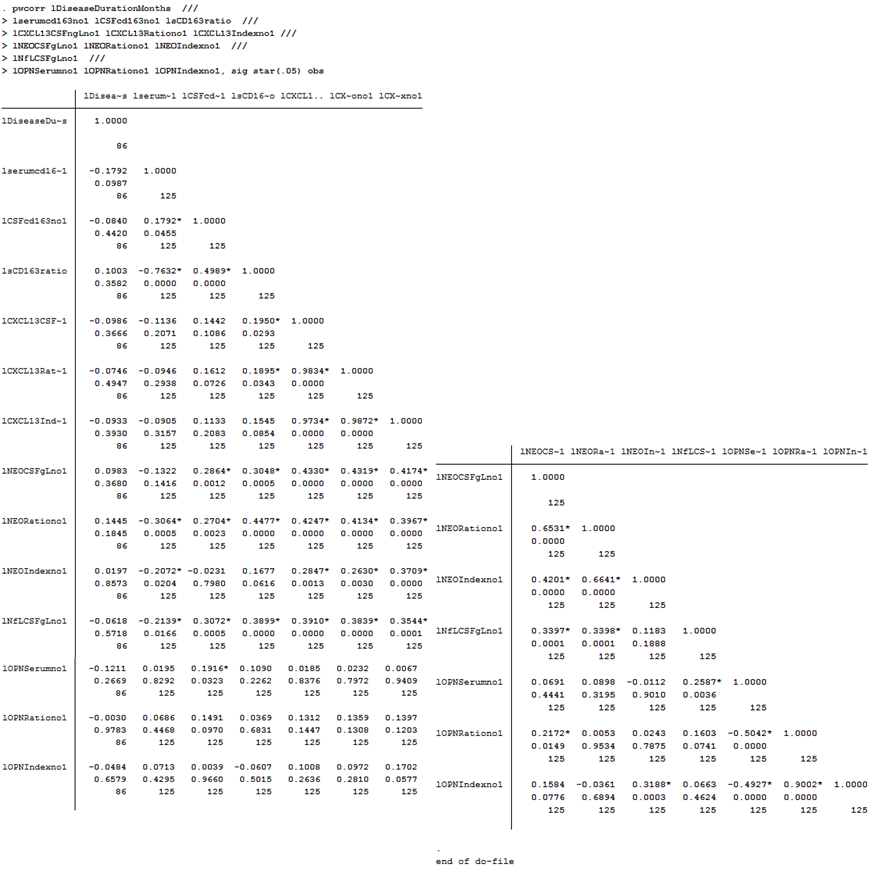
**

**Table O. Do-file and output of the Pearson correlation analysis with the Bonferroni correction on Disease duration (months).**

**
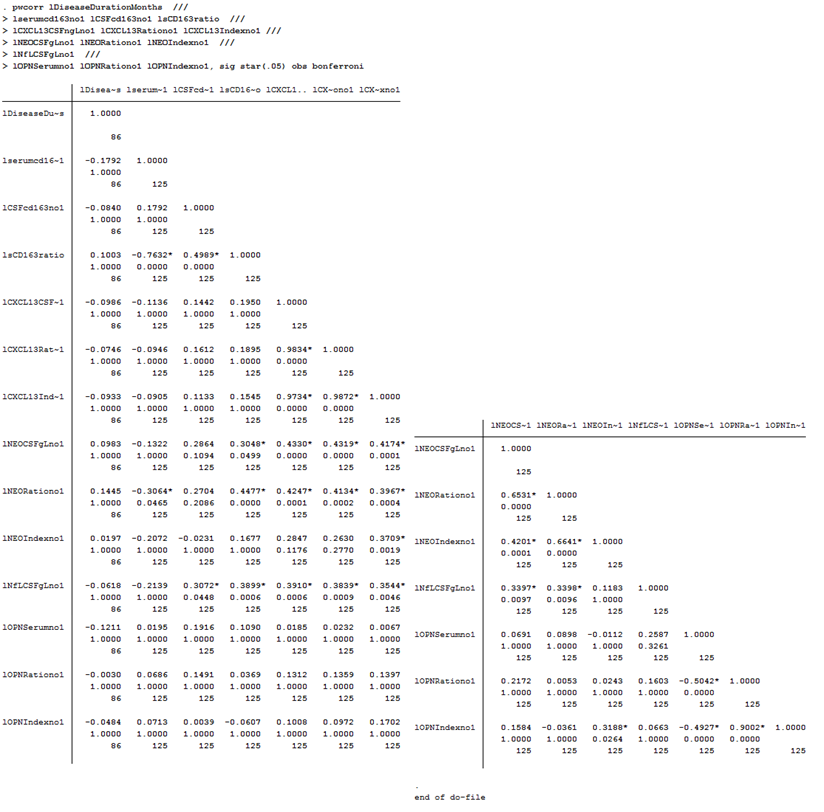
**

**Table P. Do-file and output of the Pearson correlation analysis without the Bonferroni correction on Time since last attack (days).**

**
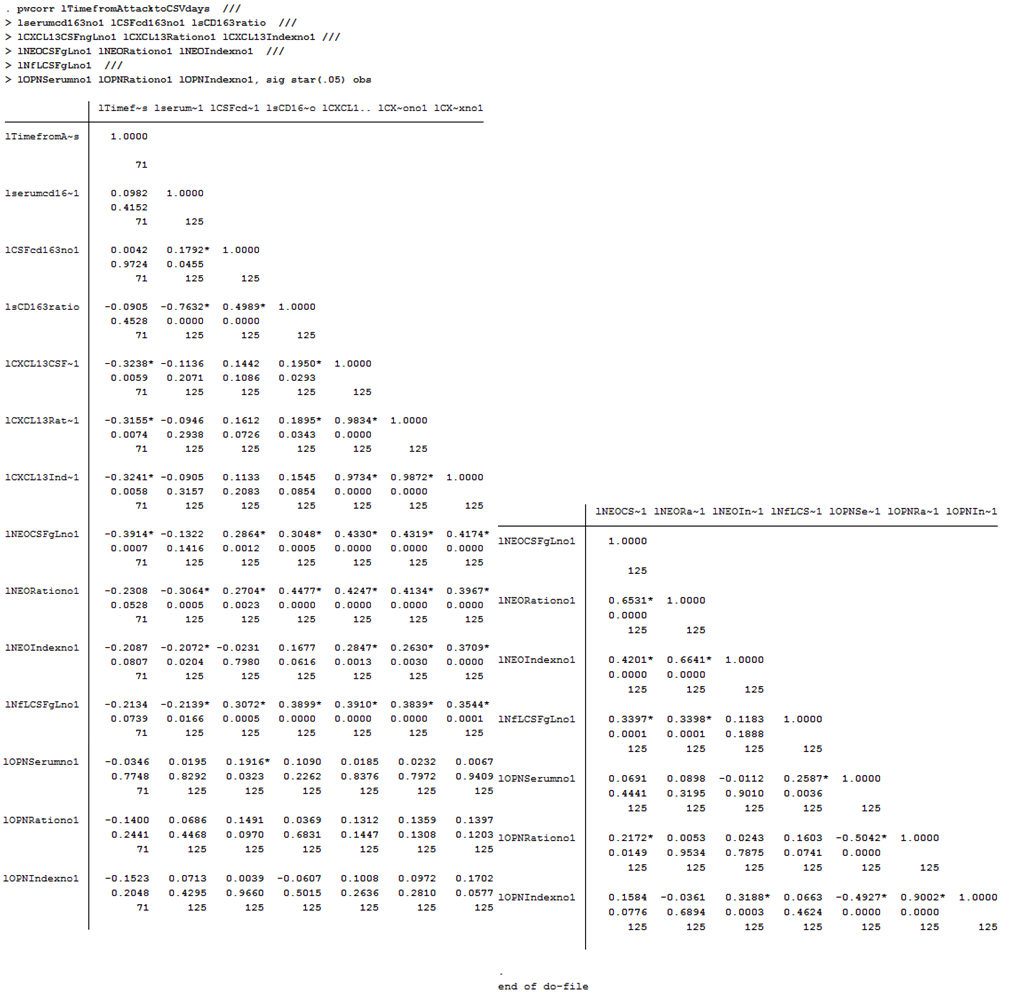
**

**Table Q. Do-file and output of the Pearson correlation analysis with the Bonferroni correction on Time since last attack (days).**

**
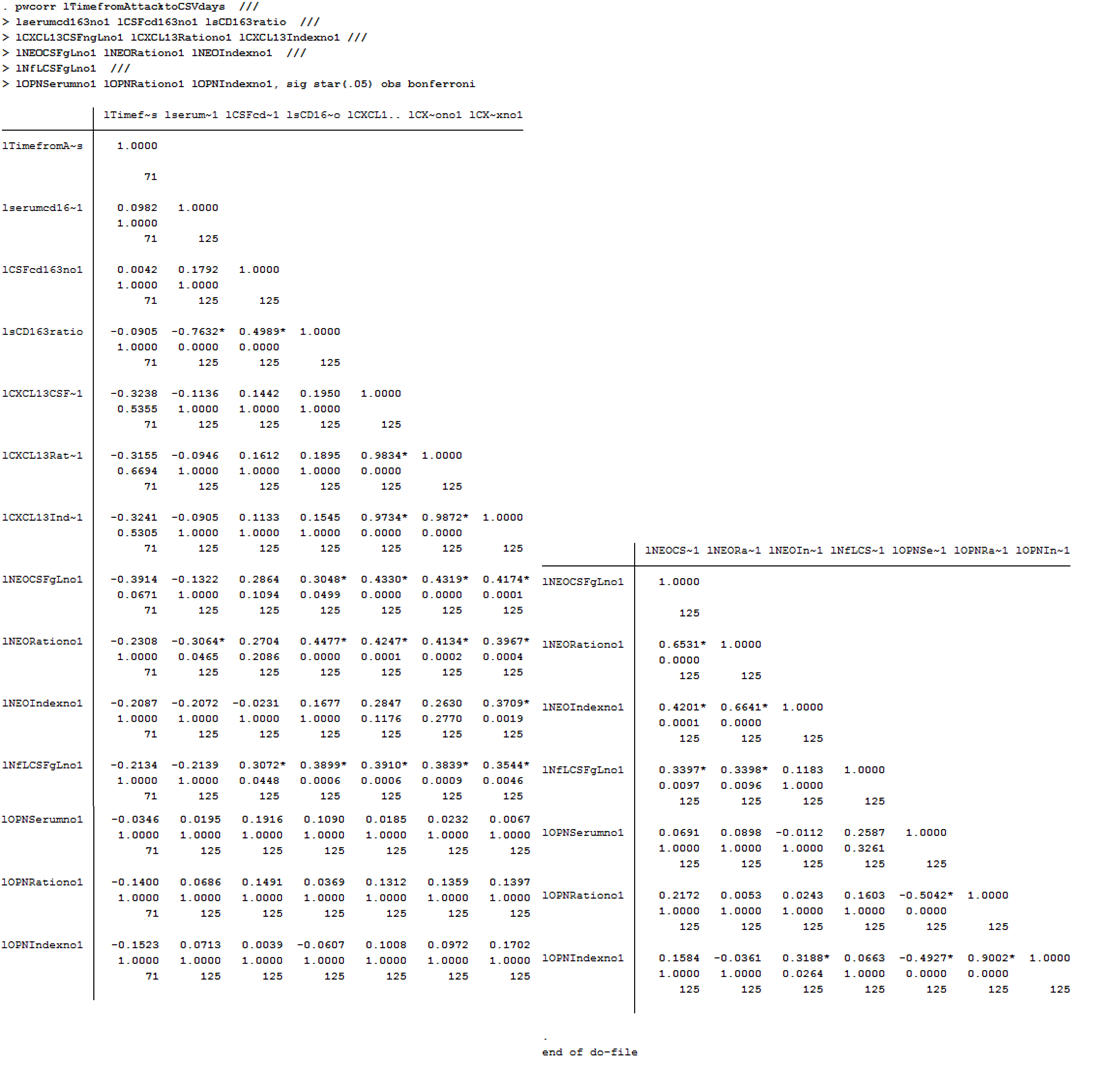
**

**Table R. Do-file and output of the Pearson correlation analysis without the Bonferroni correction on CSF Protein.**

**
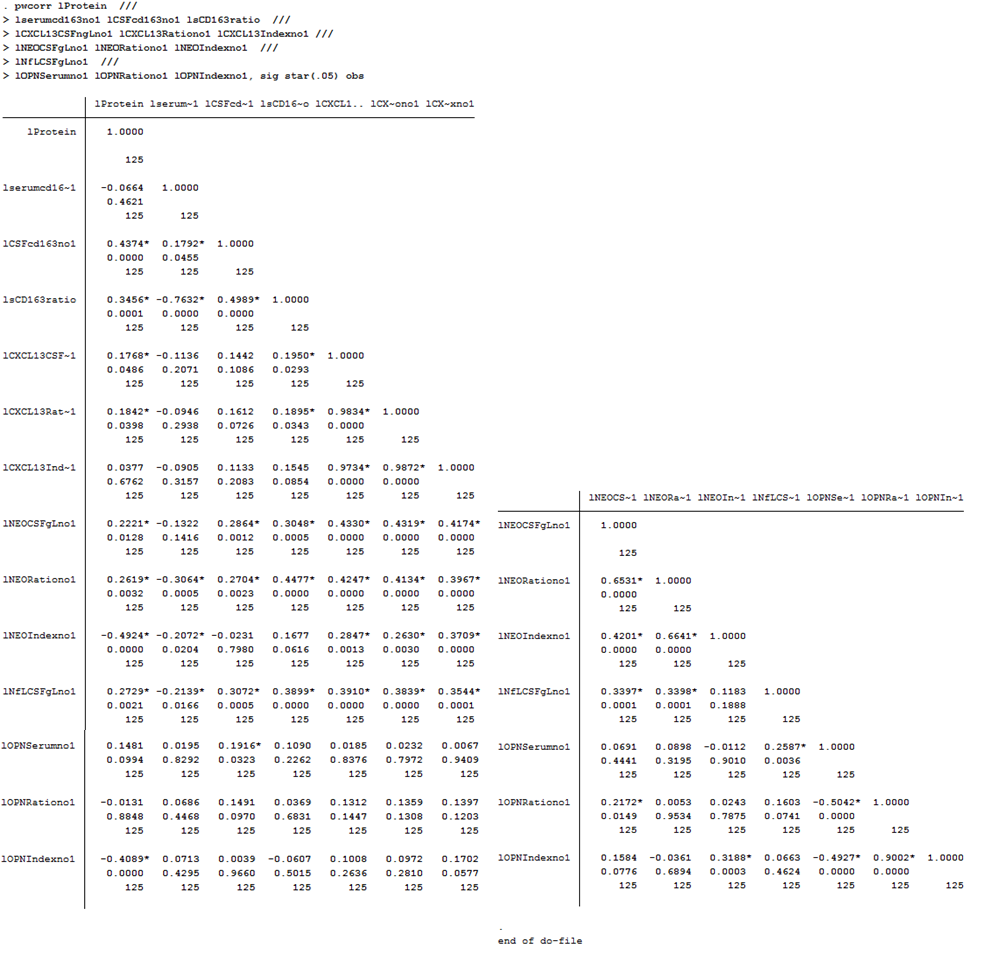
**

**Table S. Do-file and output of the Pearson correlation analysis with the Bonferroni correction on CSF Protein.**

**
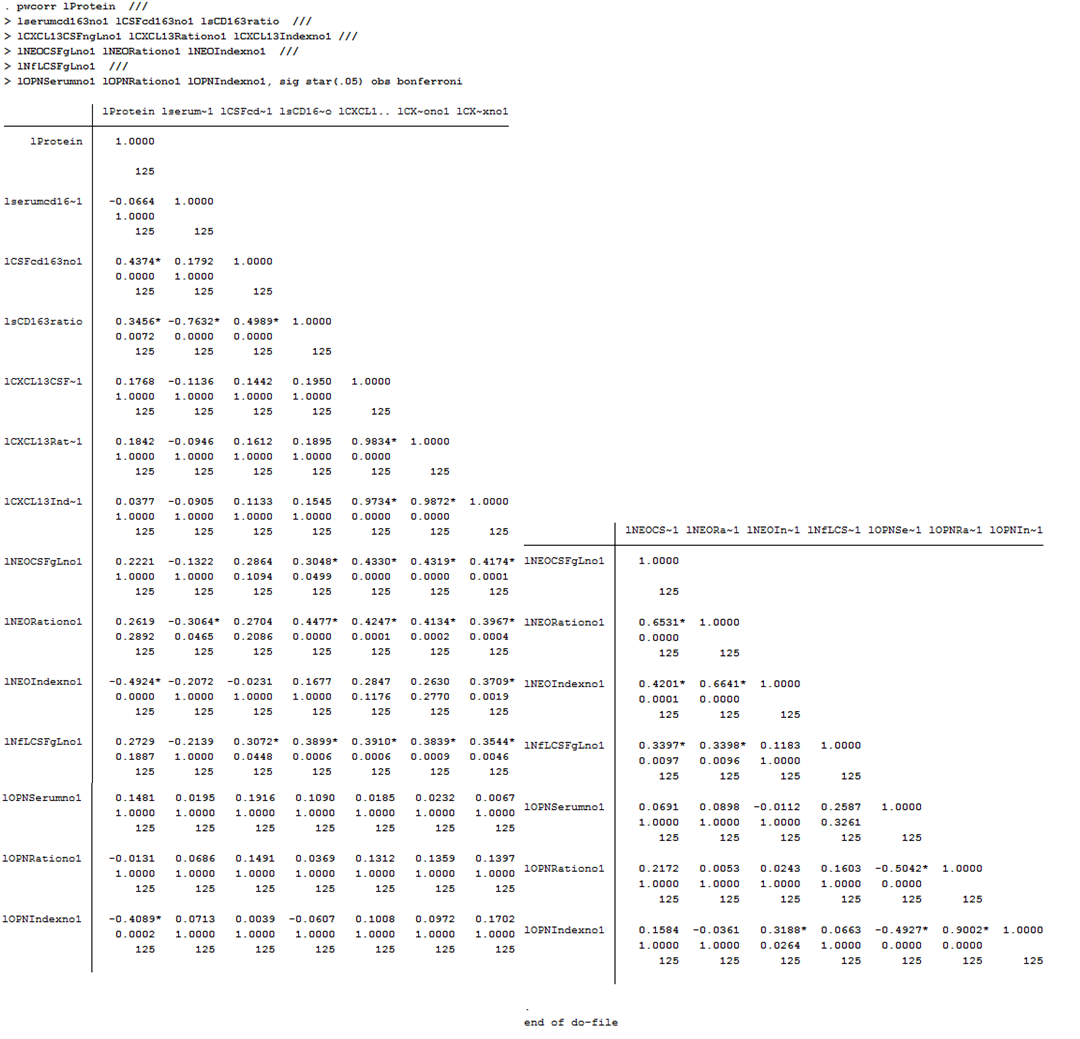
**

**Table T. Do-file and output of the Pearson correlation analysis without the Bonferroni correction on CSF Cells.**

**
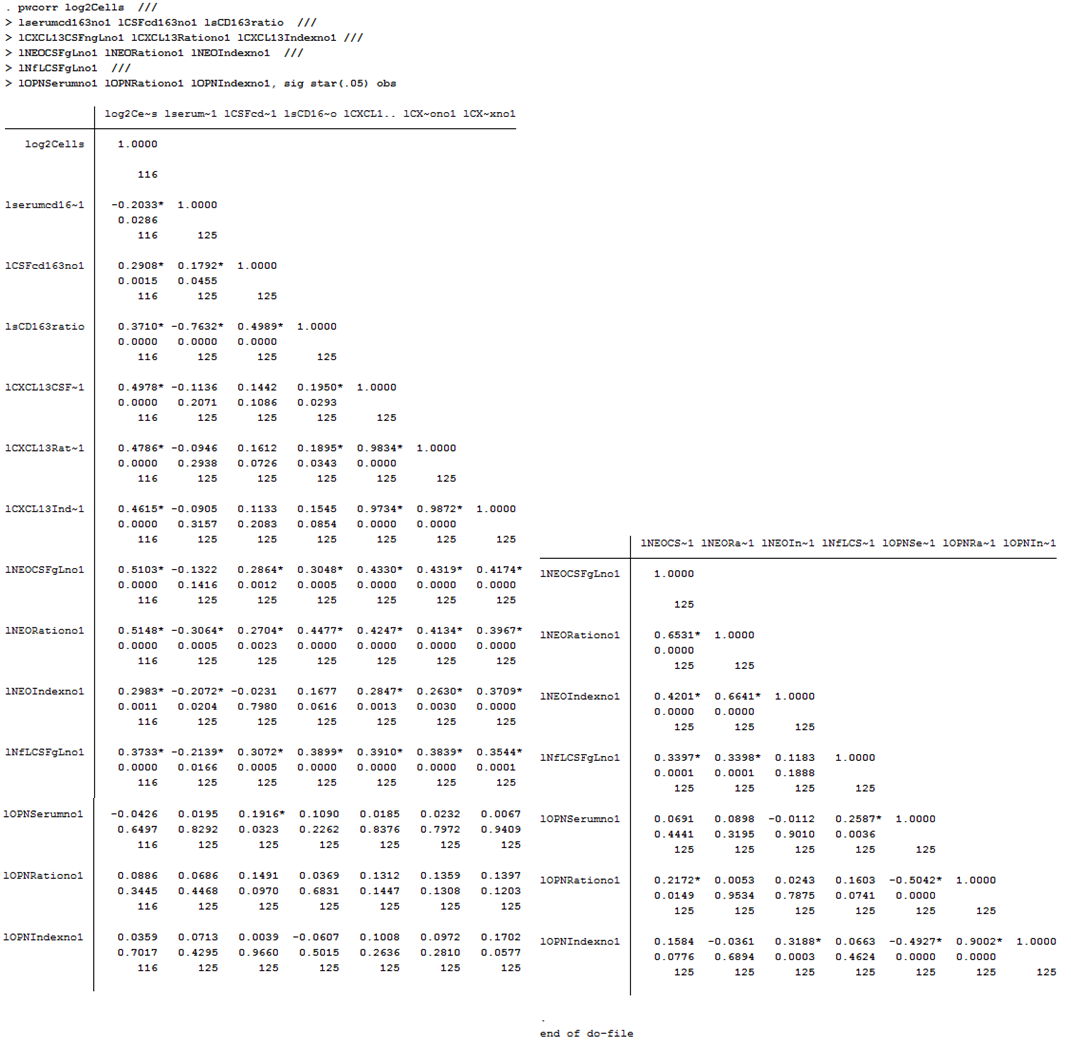
**

**Table U. Do-file and output of the Pearson correlation analysis with the Bonferroni correction on CSF Cells.**

**
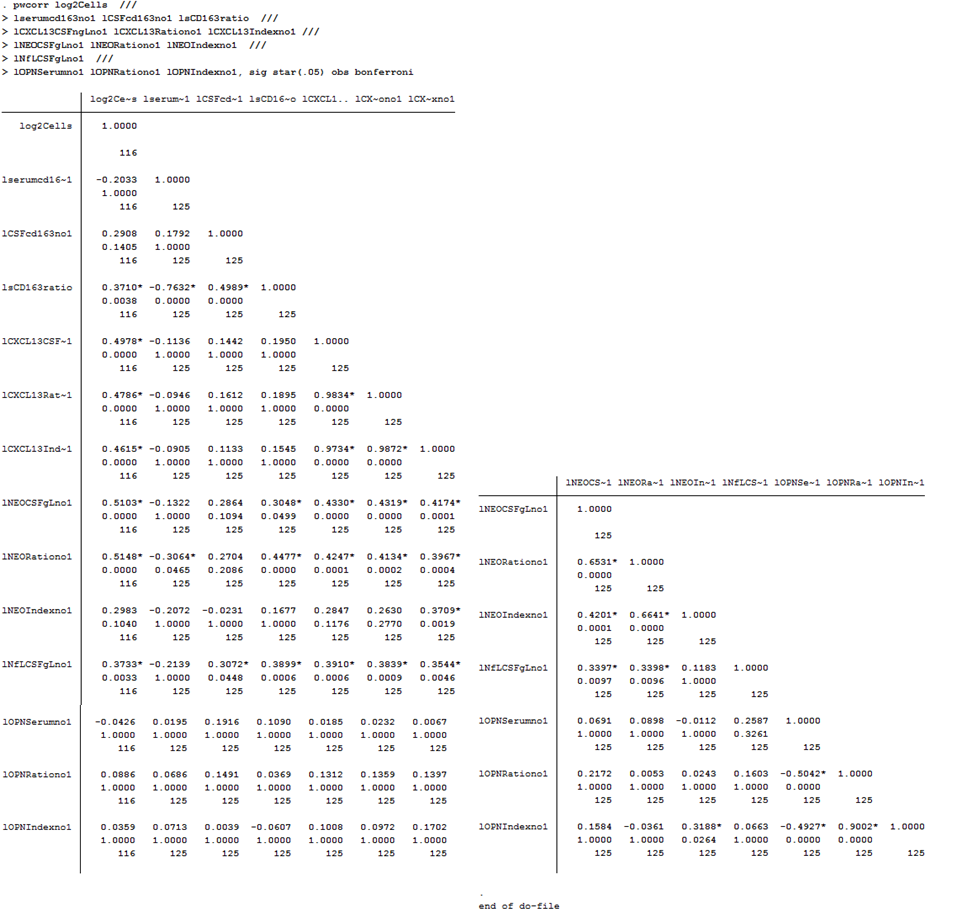
**

**Table V. Do-file and output of the Pearson correlation analysis without the Bonferroni correction on the IgG index.**

**
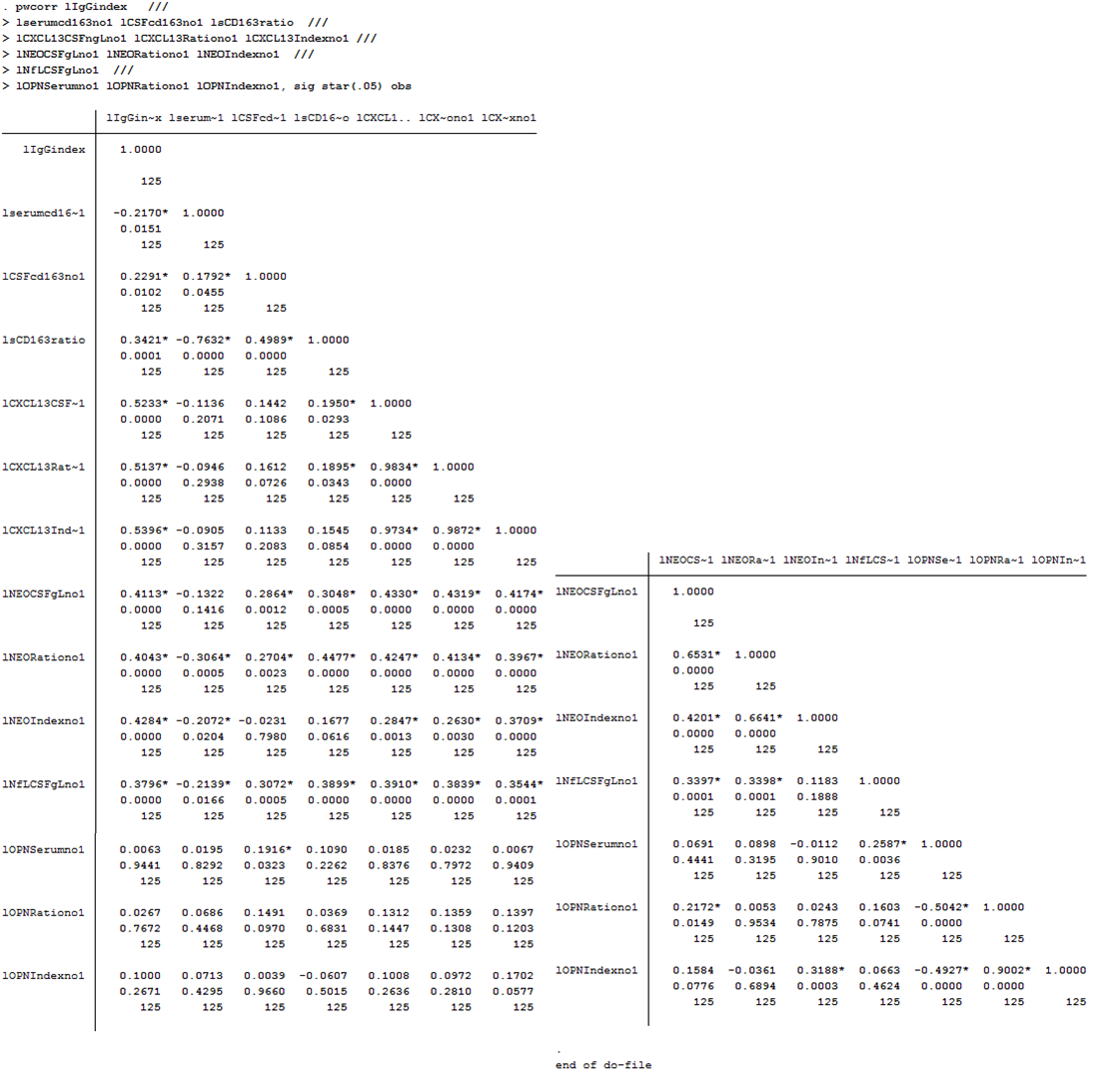
**

**Table W. Do-file and output of the Pearson correlation analysis with the Bonferroni correction on the IgG index.**

**
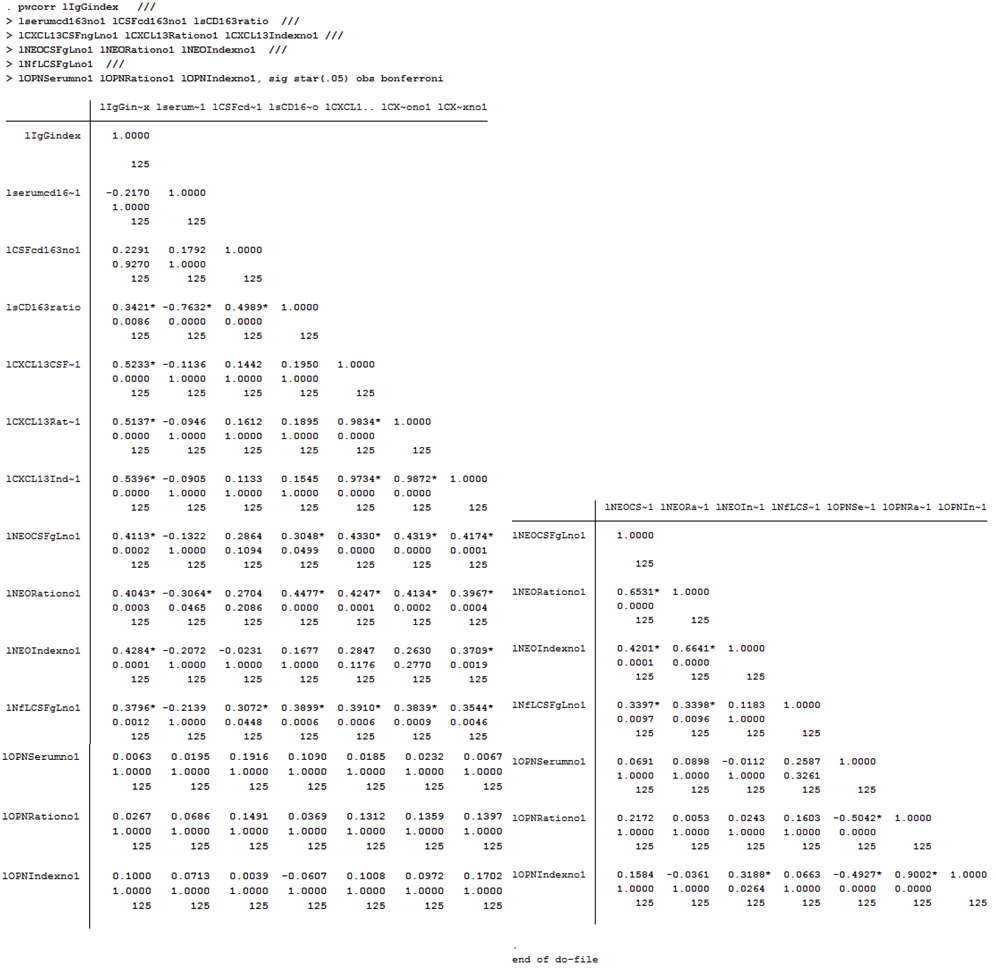
**

**Table X. Do-file and output of the Pearson correlation analysis without the Bonferroni correction on the Total number of MRI white matter lesions.**

**
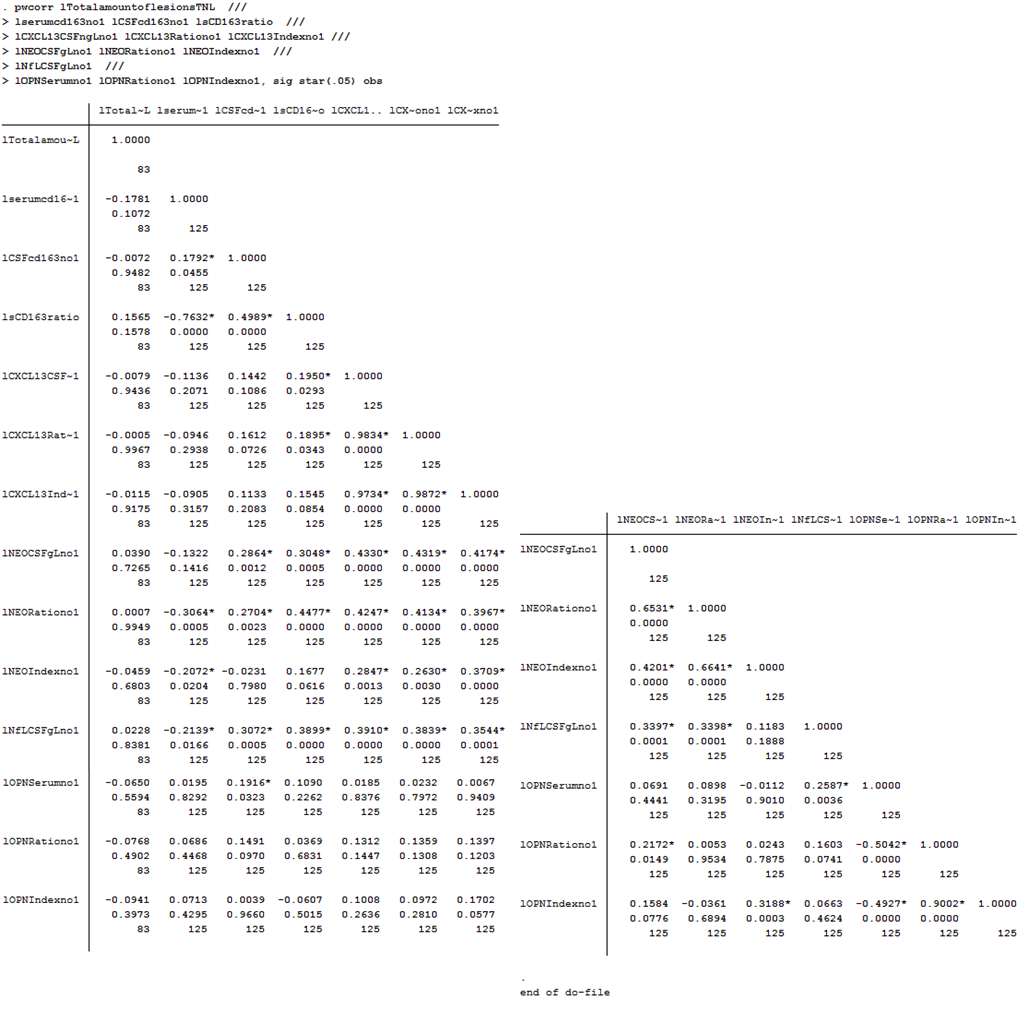
**

**Table Y. Do-file and output of the Pearson correlation analysis with the Bonferroni correction on the Total number of MRI white matter lesions.**

.

**
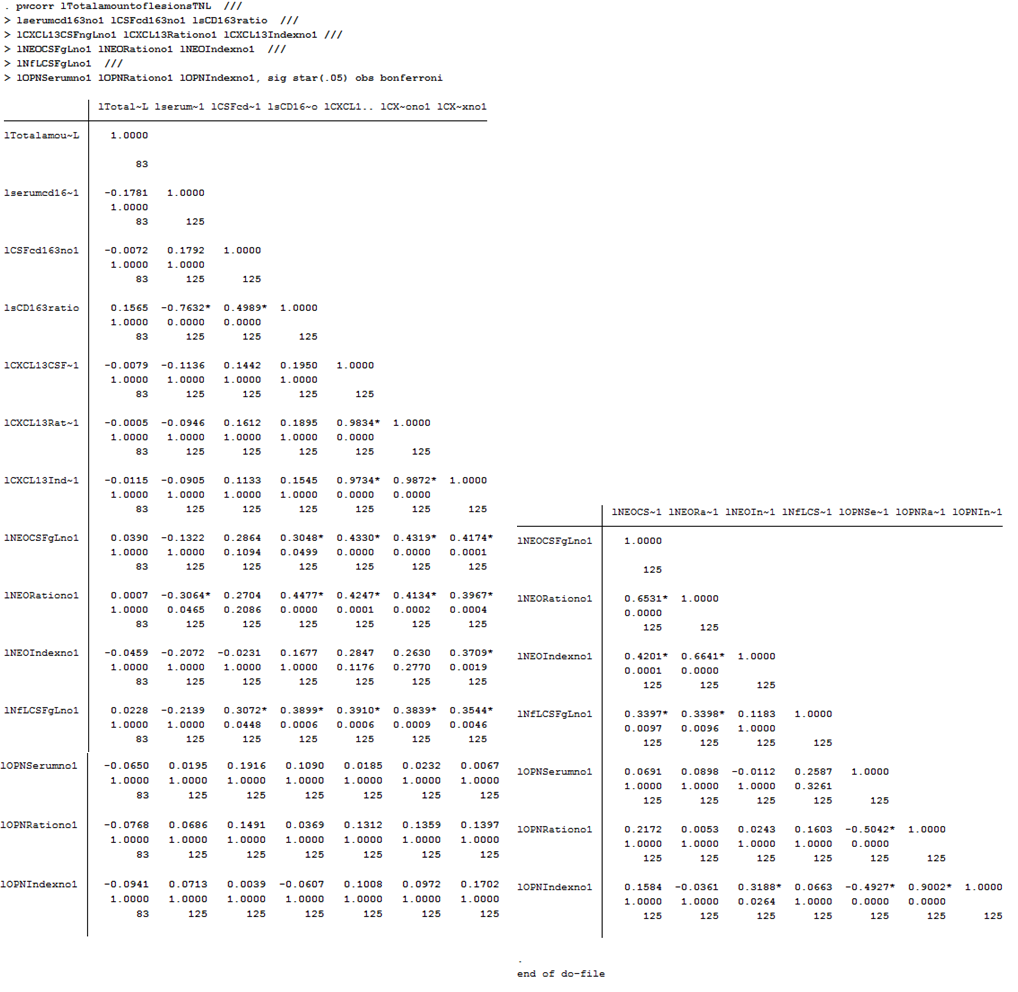
**

**Table Z. Do-file and output of the Pearson correlation analysis without the Bonferroni correction on the biomarker intercorrelations.**

**
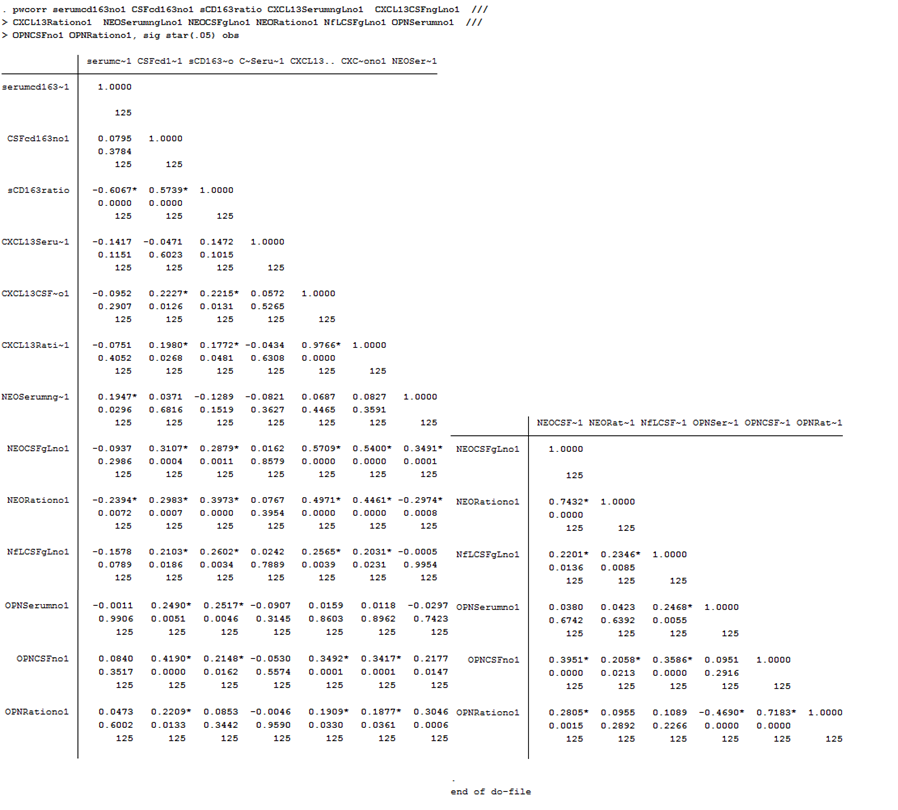
**

**Table AA. Do-file and output of the Pearson correlation analysis with the Bonferroni correction on the biomarker intercorrelations.**

**
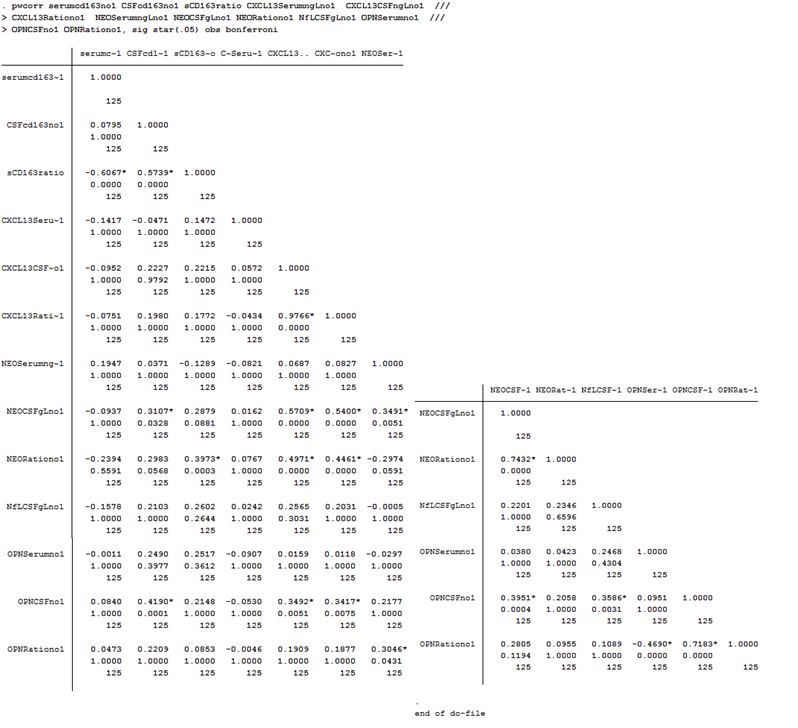
**

**Do-file and output for the regression analyses in STATA.**

The regression analysis was performed as described in our previously published paper [18] with the details for the STATA methods as reported in [60].

**Table AB. Example of do-file and output of the regression analysis on log transformed sCD163 CSF/serum values (lsCD163ratio).**


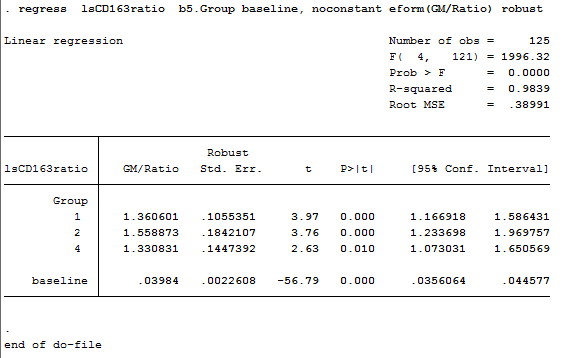


**Do-file and output for the ROC analyses in STATA.**

**Table AC. Do-file and output of the ROC analyses .**


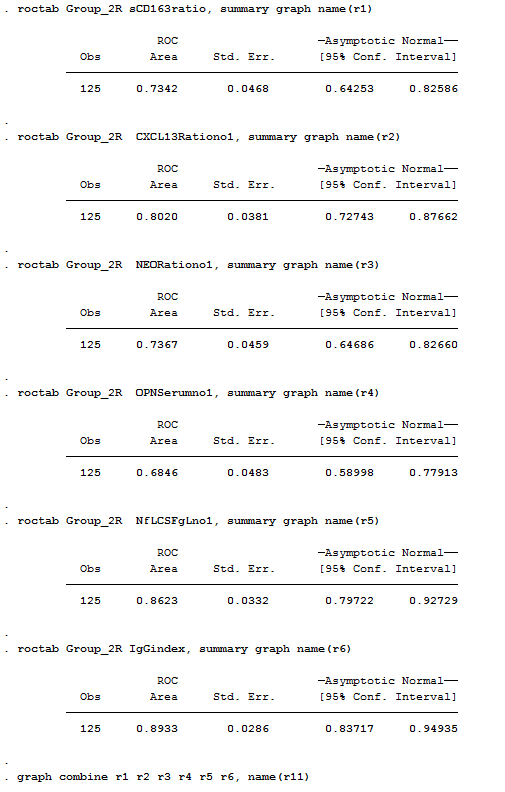


**Do-file and output for the logistic regression analyses in STATA.**

**Table AD. Example of do-file and the final output from a logistic regression analysis**


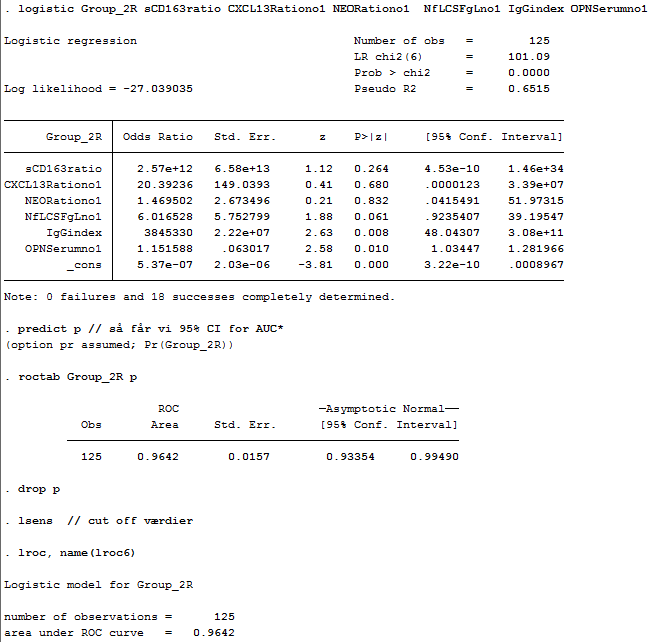


All references in S1_Dataset are given in the article.

**Contact**

For further information please contact [mortenleifms@gmail.com](mailto:mortenleifms@gmail.com)
