# Supplementary material for: Biomarkers of Inflammation and Axonal Degeneration/Damage in Patients with Newly Diagnosed Multiple Sclerosis: Contributions of the Soluble CD163 CSF/Serum Ratio to a Biomarker Panel
Source: PLoS One. 2015 Apr 10;10(4):e0119681. doi: 10.1371/journal.pone.0119681 (PMC4393241; doi:10.1371/journal.pone.0119681)
Supplement: S1 Previous Publication — (PDF) [file pone.0119681.s002.pdf]

PLOS | ONE

Soluble CD163 as a Marker of Macrophage Activity in Newly Diagnosed Patients with Multiple Sclerosis

Morten Stilund[1,3]*, Ann-Kathrin Reuschlein[2], Tove Christensen[3], Holger Jon Møller[2], Peter Vestergaard Rasmussen[1], Thor Petersen[1]

1 Department of Neurology, Aarhus University Hospital, Aarhus C, Denmark, **2** Department of Clinical Biochemistry, Aarhus University Hospital, Aarhus C, Denmark, **3** Department of Biomedicine, Aarhus University, Aarhus C, Denmark

Abstract

Background: Soluble CD163 (sCD163) is a macrophage specific protein known to be up-regulated in serum from patients with multiple sclerosis (MS).

Objective: To investigate sCD163 in serum and CSF (cerebrospinal fluid) from patients undergoing MS diagnostic work-up and analyse its potential as a diagnostic biomarker.

Methods: After a full MS diagnostic work-up, including collection of paired samples of CSF and serum, 183 patients were evaluated for inclusion in this study. Patients were divided into groups based on their diagnosis. Patients with normal clinical and paraclinical findings were grouped as symptomatic controls. Serum and CSF levels of sCD163 were determined by enzyme-linked immunosorbent assay (ELISA).

Results: sCD163 could be measured in all serum and CSF samples. A high sCD163 CSF/serum ratio in relation to molecular weight was found, strongly indicating local production in the CNS. Median levels of sCD163 were significantly decreased in serum and significantly elevated in CSF in patients with relapsing-remitting, and primary- progressive MS. There were, however, some overlaps of the measures between groups. In a receiver operating characteristic (ROC) analysis sCD163 CSF/serum ratio had an area under the curve of 0.72.

Conclusion: The sCD163 CSF/serum ratio was significantly increased in patients with MS and may reflect macrophage activation in MS lesions. These results suggest that primary progressive MS also is driven by inflammation in which the innate immune system plays a pivotal role.

Citation: Stilund M, Reuschlein A-K, Christensen T, Møller HJ, Rasmussen PV, et al. (2014) Soluble CD163 as a Marker of Macrophage Activity in Newly Diagnosed Patients with Multiple Sclerosis. PLoS ONE 9(6): e98588. doi:10.1371/journal.pone.0098588

Editor: Serge Nataf, University of Lyon, France

Received January 12, 2014; **Accepted** May 5, 2014; **Published** June 2, 2014

Copyright: © 2014 Stilund et al. This is an open-access article distributed under the terms of the Creative Commons Attribution License, which permits unrestricted use, distribution, and reproduction in any medium, provided the original author and source are credited.

Funding: This study was funded by The Danish Multiple Sclerosis Society. Grant number(s): 8990 and 14567. Funder URL: http://scleroseforeningen.dk/. The funders had no role in study design, data collection and analysis, decision to publish, or preparation of the manuscript.

Competing Interests: The authors have declared that no competing interests exist.

* E-mail: mortenleifms@gmail.com

Introduction

Multiple sclerosis (MS) is the most common, debilitating immune-mediated disease of the central nervous system in young adults. The cause of the disease is unknown, yet it is generally assumed to be a complex interaction of genetic susceptibility and environmental factors [1–3]. MS pathology is characterised by lesions in the central nervous system. These MS lesions, plaques, are infiltrated by immune cells and constitute foci of distinct patterns of demyelination, inflammation, axonal damage, and gliosis [4,5]. The underlying mechanism is currently thought to be an autoimmune dysfunction involving both the adaptive and the innate immune system [6,7].

Macrophages are abundantly present in inflammatory MS lesions [4]. Macrophages are tissue specific monocytes with versatile roles including phagocytosis, antigen presentation and lymphocyte stimulation. They orchestrate the inflammatory response in a balanced manner with flexible phenotypic responses to local environmental signals. It has also been demonstrated that macrophages in both stages of activation (pro-inflammatory (M1), and anti-inflammatory and growth promoting (M2)) are present in active MS lesions [8,9].

CD163 is a receptor for haptoglobin - hemoglobin complexes and a monocyte/macrophage specific membrane protein. It is found on macrophages in the CNS, including perivascular macrophages (PVMs) and microglia [10]. Membrane bound CD163 (mCD163) is considered a marker for the M2 pathway, although mCD163 is also up-regulated in chronically inflamed tissues [11–13].

CD163 can be found in a soluble form (sCD163) in plasma and other body fluids such as cerebrospinal fluid (CSF) [14]. In the CNS, sCD163 is probably shed by macrophages and microglia, triggered by complex immune modulating mechanisms in the microenvironment. In a study of 129 random blood samples, mCD163 has been shown to be inversely related to the plasma levels of sCD163 [15] and in another study proteolytic shedding of

CD163 has been shown to be increased in serum of MS patients [16].

sCD163 can be measured in serum by enzyme linked immune-sorbent assay (ELISA) [17], and changes in sCD163 has been associated with a number of inflammatory disorders, as recently reviewed [18].

In this study we aimed to investigate whether sCD163 is measurable in CSF and serum of newly diagnosed MS patients. We wanted to estimate the intrathecal production of sCD163 and investigate putative diagnostic qualities of sCD163 in a large cohort of patients with newly diagnosed remitting relapsing MS (RRMS), primary progressive MS (PPMS), secondary progressive MS (SPMS), clinically isolated syndrome (CIS), and compare with a symptomatic control (SC) group [19].

Materials and Methods

Ethics statement

The study was conducted in accordance with the Ethical Declaration of Helsinki and all patients gave written, informed consent as approved by The Central Denmark Region Committee on Biomedical Research Ethics (journal number: 20090210)

Patients

From September 2009 to December 2011 patients admitted for further diagnostic investigations to the MS clinic, at the Department of Neurology, Aarhus University Hospital, were consecutively included in the study. Diagnostic procedures consisted of anamnesis, clinical examination, MRI of the entire neural axis, CSF analyses (cells, protein, immunoglobulin G (IgG) index), and evoked potentials as recommended [20]. EDSS (extended disability status scale) was assessed according to Kurtzke [21]. MRI and cerebrospinal fluid examination were evaluated according to the latest classification of MS [20].

Patient demographics and clinical data are presented in Table 1. A total of 183 patients agreed to participate and serum and CSF samples were collected and frozen at −70°C for later examination in accordance with consensus guidelines [22].

The inclusion criteria were met by 130 patients and the inclusion and exclusion of patients are illustrated in Figure 1. Patients were excluded if they had other neurologic disease, a medical disease, had been undergoing immune modulating treatment within the month preceding sampling, had paraclinical isolated syndromes, or if data were missing. Patients included in the study cohort were divided into five subgroups: RRMS (n = 45), PPMS (n = 15), SPMS (n = 4)), CIS (n = 27) and, SC (n = 39) with either normal or unspecific MRI. For further details on exclusion please see Data S1.

The diagnoses were established before the results of sCD163 analysis were received.

ELISA for sCD163 measurement in serum and CSF

sCD163 was measured by ELISA essentially as described [17]. Briefly, rabbit anti-CD163 (2 mg/L, SK Moestrup Aarhus University) was coated onto microtitre wells. Serum samples (diluted 1:101) or CSF samples (diluted 1:4) was added in duplicates and incubated for 1 h at RT. Monoclonal anti-CD163 (clone number: GHI/61,3 μg/mL) was added followed by incubation for 1 h at RT with horseradish peroxidase-labelled goat anti-mouse antibodies (0.125 μg/mL; Dako, Glostrup, Denmark). The assay was calibrated using serum traceable to purified human CD163, with the lowest calibrator being 6.25 μg/L.

Validation of ELISA for CSF measurements

In a pilot experiment, results were compared with a commercial sCD163 assay (Macro163TM, IQ-products Groningen NL), showing good correlation, however with a bias due to lack of assay standardisation as described earlier for serum samples [18]. The intra-assay precision was determined in two pools of cerebrospinal fluid with low and high sCD163 concentrations. The intra-assay precision (coefficient of variation) was 3.0% at a level of 0.123 mg/L (n = 24) and 7.9% at a level of 0.073 mg/L (n = 24). The total imprecision was determined from diluted serum-control samples included in each plate and ranged from 5.3–12.9% (n = 13). Linearity was confirmed by measuring a diluted (2, 4, 8, and 16 times) pool of CSF in quadruplicate. Aliquots of pools of samples were kept at −80°C and showed stable concentrations for >1 year.

Collection of data and statistical analysis

All medical history, and biochemical data on patients were collected using the Electronic Patient Journal (EPJ) and radiological data was collected via written descriptions of MRIs by a senior neuroradiologist and confirmed by a senior neurologist who viewed the scans in the IMPAX system at the Department of Neurology, AUH. Data was stored and handled according to the Danish law on personal data.

We used Microsoft Excel for data collection and statistical analysis was performed using STATA12. The CSF and Serum values were not normally distributed and, therefore, we performed all our analyses on log-transformed values.

For group comparison we performed simple linear regression analysis with age adjustment for each parameter. The age adjusted geometric means (GM) of the SC group were used as baseline denominators to produce ratios with the geometric means for RRMS, PPMS, and CIS as integers. These ratios are expressed in percentages and levels of significance are shown by p-values [23].

All ratios (i.e. sCD163 and albumin ratio) were derived by simply dividing the CSF concentration by the serum concentration. The sCD163 index was calculated by dividing sCD163 ratio by albumin ratio. The percentage of intrathecally produced sCD163 was calculated as according to Galea et al [14]. We used two previously published methods [14,24] to evaluate the intrathecal production of sCD163. The sCD163 produced intrathecally was calculated by this formula: serum sCD163 x (CSF/serum albumin ratio) subtracted from the absolute CSF concentration. The result is presented as a percentage of the absolute CSF concentration. The ROC (receiver operating characteristic) analyses was performed for sCD163 CSF, sCD163 serum, sCD163 CSF/serum ratio, and sCD163 index and compares the MS and CIS groups with the SC group.

Results

Measurement of sCD163 by ELISA

Soluble sCD163 was detectable in CSF and serum in all individuals (Figure 2a and 2b, Table 2). For the SC group the median level of sCD163 in serum was 1.70 mg/L (range 0.97–3.73 mg/L). This corresponds well with the sCD163 serum reference interval for healthy subjects (0.7–3.9 mg/L) [18]. The median level of sCD163 in CSF for the SC group was 0.076 mg/L as compared with 1.70 mg/L in serum, yielding a median CSF/serum ratio of 0.041. We calculated that ~87% of sCD163 CSF, in the SC group, was produced intrathecally. The sCD163 CSF/serum ratio and sCD163 Index values are shown in Figure 2c and 2d. Figures 2a–2d show some overlaps in levels between patient groups, and symptomatic controls.

Table 1. Demographic data of the patients with MS/CIS and SC

Characteristics	RRMS	PPMS	SPMS	CIS	SC
**No. of Subjects (total = 130)[a]**	n = 45	n = 15	n = 4	n = 27	n = 39
**Gender M/F**	8/37	8/7	2/2	7/20	4/35
**Mean Age[b]**	37	53	52	37	41
**(range)**	(23–62)	(35–64)	(45–57)	(16–71)	(25–57)
**Mean EDSS**	2.3	3.7	3.1	1.7	0
**(range)**	(0–4.0)	(2.0–7.0)	(2.0–4.0)	(0–3.0)	
**Mean No. of attacks[c]**	2.91	0	1.20	1	N/A
**Disease Duration**	56.5	55	333	18.5	0
**(range)[d]**	(3.5–288)	(14–228)	(240–444)	(0.5–90)	
**Time since last attack**	129 d	N/A	22 y	388 d	N/A
**(range)[e]**	(1–720)		(9–37)	(3–2745)	
**Mean CSF Protein, μmol/L**	0.38	0.40	0.44	0.39	0.33
**(range)**	(0.2–0.63)	(0.2–0.65)	(0.31–0.52)	(0.16–0.74)	(0.20–0.57)
**Mean CSF Cells, 10^6/L**	9.8	5.6	4.5	8.11	1.6
**(range)**	(0–39)	(1–12)	(1–8)	(0–55)	(0–5)
**Mean IgG Index**	1.17	1.12	1.02	0.90	0.49
**(range)**	(0.44–3.04)	(0.44–3.94)	(0.47–1.76)	(0.42–2.81)	(0.40-0-62)
**Mean total number of MRI white matter lesions**	18	18	32	9	0
**(range)**	(5–55)	(2–45)	(9–55)	(0–42)	

Table 1 shows the demographics of the patient cohort.
[a]Refers to the patients included, see figure 1.
[b]Age (in years) refers to age at sampling time point. [c]Mean number of attacks, refers to attacks before sampling time point.
[d]Disease duration (in months) refers to period from debut of first symptom(s) to sampling time point.
[e]Time since last attack, refers to the period (days = d or years = y) from latest attack to sampling time point. Abbreviations: RRMS (relapsing-remitting MS), PPMS (primary-progressive MS), SPMS (secondary-progressive MS), CIS (clinically isolated syndrome), SC (symptomatic controls), N/A (not applicable or available), n (number of persons), EDSS (Expanded Disability Status Scale). CSF (cerebrospinal fluid).
doi:10.1371/journal.pone.0098588.t001

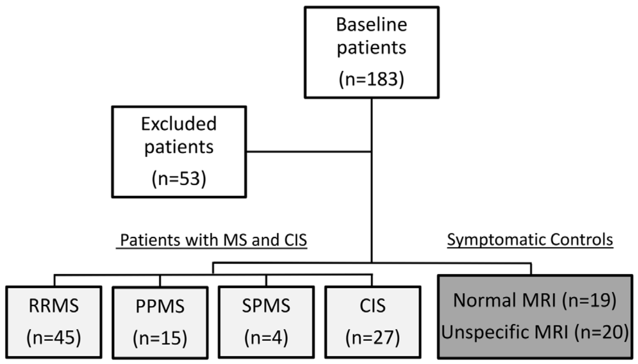

Figure 1. Flowchart demonstrating exclusion and inclusion criteria for the study. Patients were excluded if they had other neurologic disease, a medical disease, had been undergoing immune modulating treatment within the month preceding sampling, had paraclinical isolated syndromes, or if data were missing. Patients included in the study cohort were divided into five subgroups: relapsing-remitting MS (RRMS), primary progressive MS (PPMS), secondary progressive MS (SPMS), clinical isolated syndrome (CIS) and, symptomatic controls (SC) with either normal or unspecific MRI. Abbreviations: RRMS (relapsing-remitting MS), PPMS (primary-progressive MS), SPMS (secondary-progressive MS), CIS (clinically isolated syndrome), SC (symptomatic controls), CSF (cerebrospinal fluid), CI (confidence interval).
doi:10.1371/journal.pone.0098588.g001

Correlation analysis of sCD163 measurements and demographic data

Patients in the PPMS and SPMS groups were significantly older than patients in the RRMS, CIS and SC groups; and levels of sCD163 in both serum and CSF were found to correlate with age. Consequently, all regression analyses were performed on data adjusted for age as described in the statistical analysis section.

The levels of sCD163 in serum and CSF were not correlated to gender or any of the clinical measures: time since last attack, EDSS, mean no. of relapses, mean disease duration, enhancing lesions or the total number of MRI white matter lesions (TNL). TNL and EDSS were correlated to group as patients with CIS had fewer lesions and lower EDSS than patients with RRMS and PPMS.

Age adjusted linear regression

Results from the age adjusted linear regression analysis are presented in Table 3 and in the Data S1. Interestingly, the age adjusted geometric means (GM) of sCD163 in CSF were significantly elevated for patients in the RRMS ($p = 0.026$), PPMS ($p = 0.011$), and CIS ($p = 0.014$) groups compared with the SC group. In serum, the GM levels of sCD163 were significantly lower in the RRMS ($p = 0.006$) and PPMS ($p = 0.008$) groups compared with the SC group. The GM values of the sCD163 CSF/serum ratio was significantly increased in RRMS ($p < .001$), CIS ($p = 0.011$), and especially the PPMS group in which the GM value was elevated by 56% ($p < 0.001$). The GM value of sCD163 index was significantly elevated by 41% ($p = 0.022$) in the PPMS

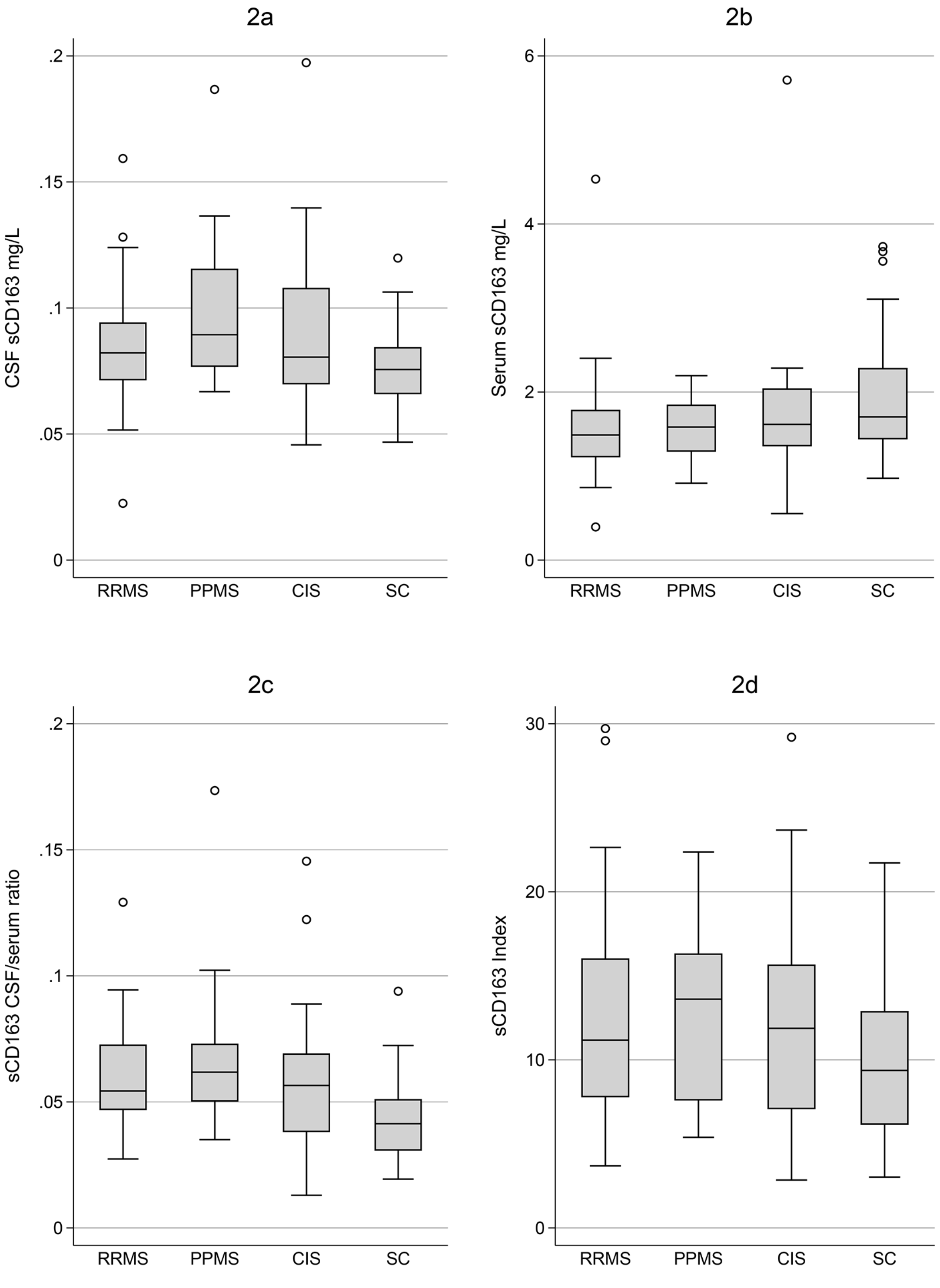

Figure 2. Box plots of sCD163 concentrations, ratios, and indexes. Four graphs (2a–2d) with levels of: sCD163 in CSF (2a); serum (2b); the sCD163 CSF/serum ratios (2c); and the sCD163 indexes (2d). The box plots displays the distribution of the parameters for each group, RRMS, PPMS; SPMS; CIS and SC. The line in the box indicating the median level, whiskers fences the upper and lower quartiles, and "o" labels the outliers. Abbreviations: RRMS (relapsing-remitting MS), PPMS (primary-progressive MS), CIS (clinically isolated syndrome), SC (symptomatic controls), CSF (cerebrospinal fluid), CI (confidence interval).
doi:10.1371/journal.pone.0098588.g002

group compared with the SC group. Due to the small number of patients with SPMS in the study cohort, this group was omitted from the figures 2a–2d and also from the linear regression analysis.

Receiver Operating Curve analysis of sCD163 diagnostic properties

Results of ROC analyses of sCD163 CSF, sCD163 serum, sCD163 CSF/serum ratio, and sCD163 index are shown in

Table 2. Median levels of sCD163 in serum and CSF samples as determined by ELISA.

Characteristics	RRMS	PPMS	SPMS	CIS	SC
**No. of Subjects (total = 130)**	n = 45	n = 15	n = 4	n = 27	n = 39
**Serum sCD163 mg/L**	1.49	1.58	2.0	1.62	1.70
**(range)**	(0.39–4.53)	(0.92–2.2)	(1.27–5.16)	(0.55–5.71)	(0.97–3.73)
**CSF sCD163 mg/L**	0.084	0.089	0.097	0.081	0.076
**(range)**	(0.023–0.159)	(0.067–0.187)	(0.077–0.114)	(0.046–0.197)	(0.047–0.120)
**sCD163 CSF/serum ratio**	0.054	0.062	0.048	0.057	0.041
**(range)**	(0.027–0.129)	(0.035–0.174)	(0.017–0.084)	(0.013–0.145)	(0.019–0.094)
**sCD163 index**	11.2	13.6	7.7	11.9	9.4
**(range)**	(3.7–29.7)	(5.4–22.4)	(1.9–10.1)	(2.9–29.2)	(3.0–21.7)

Table 2 shows the median levels of sCD163 in serum, CSF and, sCD163 CSF/serum ratio, and sCD163 index. Abbreviations: RRMS (relapsing-remitting MS), PPMS (primary-progressive MS), SPMS (secondary-progressive MS), CIS (clinically isolated syndrome), SC (symptomatic controls), n (number of persons), CSF (cerebrospinal fluid).
doi:10.1371/journal.pone.0098588.t002

Figure 3a–3e. Also, this figure displays a small table with area under curve (AUC) and corresponding 95% Confident Interval (CI). As a diagnostic marker the sCD163-ratio performed better than measurement in CSF alone (AUC 0.72 (95% CI: 0.63–0.81) vs. AUC 0.69 (95% CI: 0.60–0.78)). However, the IgG index was superior as a diagnostic marker to sCD163 CSF, sCD163 serum, sCD163 CSF/serum ratio, and sCD163 Index measurements with an AUC of 0.91 (95% CI: 0.86–0.95).

Discussion

Soluble CD163 could be measured in serum and CSF in all persons of the study cohort.

The intrathecal synthesis of sCD163 in the SC group of this study was more than 80% and this is in agreement with results from investigations in healthy controls [14] and places sCD163 with other molecules produced in CNS, that show a higher concentration than would be expected from the molecular weight [24]. The intrathecal production may originate from activated perivascular macrophages and microglia known to express CD163 in MS. The function of sCD163 in the CNS is unknown. A number of inflammatory stimuli, such as oxidative stress and microbial toxins are known to mediate sCD163 shedding, which in turn may modulate macrophage activation. sCD163 has also been shown directly to inhibit T-cell activation [25,26].

In patients with a demyelinating disease in the CNS we found significantly elevated levels of sCD163 in the CSF. This is in agreement with neuropathological investigations of active lesions in patients with RRMS. In these active lesions macrophages were densely present and could also be seen in chronically, active lesions [27]. In chronic inflammatory lesions, macrophages are probably activated by complex mechanisms involving both pro- and anti-inflammatory stimuli. Increased mCD163 is considered to be an anti-inflammatory response to prolonged inflammation in diseases such as macrophage activation syndrome, sepsis, and liver disease [16,28–31].

A small group of patients in the present patient cohort were diagnosed with SPMS. They were diagnosed a long time after their first relapse (15 to 38 years, see Table 1) and had slow progression of symptoms. Essentially, they represent the benign course of MS. These patients are not representative for patients with SPMS in general and were omitted from figure 2 and the linear regression analysis.

Table 3. Results from linear regression analyses of differences between patients with MS/CIS and SC.

Characteristics	RRMS	PPMS	CIS	SC
**No. of Subjects (total = 130)**	n = 45	n = 15	n = 27	n = 39
**Serum sCD163, difference in percentages from SC group**	−19%	−22%	−11%	1.49
**(p-values)**	(0.006)*	(0.008)*	(0.277)	
**CSF sCD163, difference in percentages from SC group**	12%	22%	19%	0.059
**(p-values)**	(0.026)*	(0.011)*	(0.014)*	
**sCD163 ratio, difference in percentages from SC group**	38%	56%	21%	0.040
**(p-values)**	(<0.001)*	(<0.001)*	(0.011)*	
**sCD163 Index, difference in percentages from SC group**	20%	41%	21%	11.18
**(p-values)**	(0.095)	(0.022)*	(0.171)	

Table 3 shows the results of linear regression on four different parameters: serum sCD163, CSF sCD163, sCD163 CSF/serum ratio, and sCD163 index. The regression analyses are done on log transformed, age adjusted data. Differences are shown in percentages for RRMS, PPMS, and CIS compared with the SC group. The level of significance is shown by the p-value and * marks a significant difference between groups. The SC column shows the age corrected geometric means for the SC group used in the regression analysis. Abbreviations: RRMS (relapsing-remitting MS), PPMS (primary-progressive MS), CIS (clinically isolated syndrome), SC (symptomatic controls), n (number of persons), CSF (cerebrospinal fluid).
doi:10.1371/journal.pone.0098588.t003

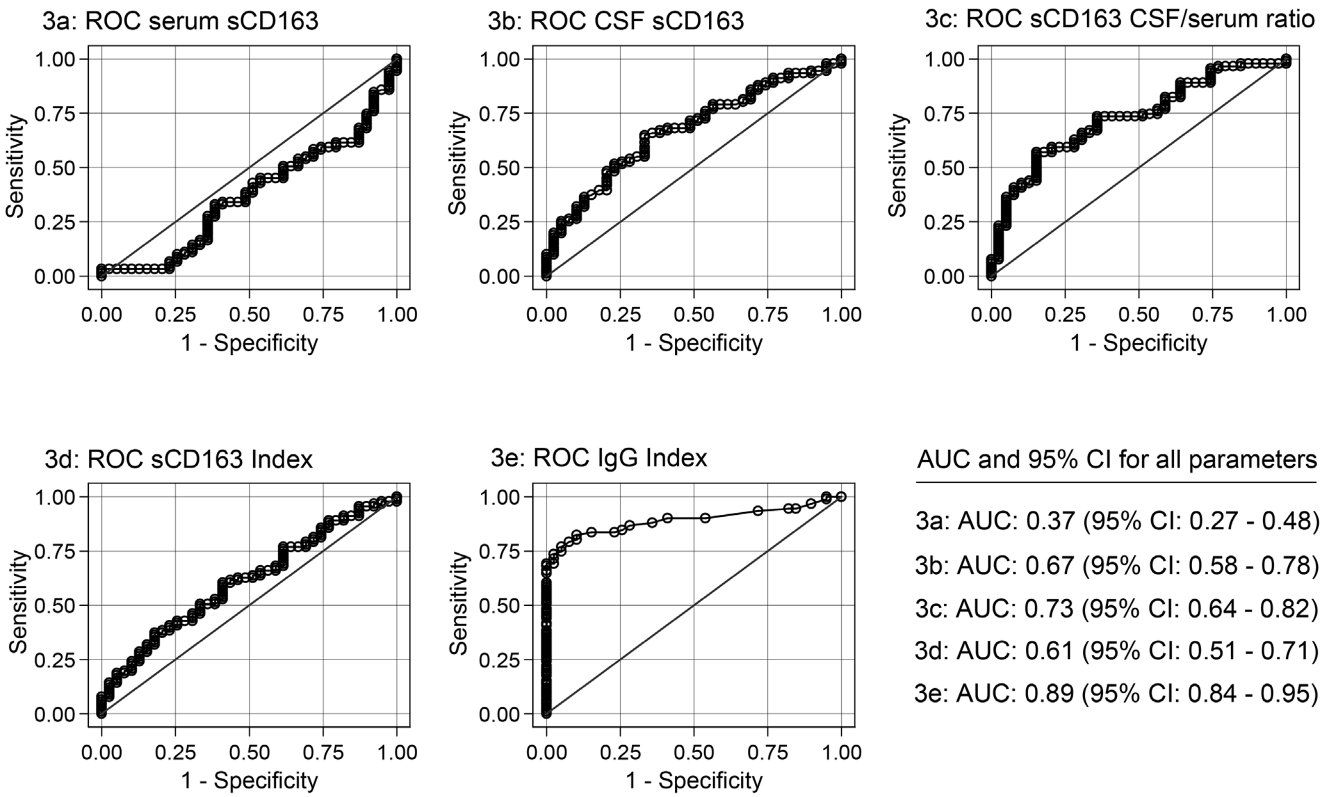

Figure 3. ROC curves generated for serum sCD163, CSF sCD163, sCD163 CSF/serum ratio, sCD163 Index, and IgG Index. AUC, with 95% CI, is given for each parameter. The parameter results for patients with MS/CIS are combined as true positives and plotted against SC as true negatives. The diagonal dividing the ROC space represents the random event. Abbreviations: ROC (receiver operating characteristic), AUC (area under the curve), CIS (clinically isolated syndrome), SC (symptomatic controls), CSF (cerebrospinal fluid), CI (confidence interval).
doi:10.1371/journal.pone.0098588.g003

In serum we found that sCD163 levels were significantly decreased when comparing patients with RRMS and PPMS with patients in the SC group. In a study by Fabriek et al sCD163 was shown to be up-regulated in MS patients when compared to healthy controls [16]. One explanation for the discrepancy is likely to be differences in the patient cohorts. The patients in the present study were newly diagnosed with MS, they had lower EDSS, and they were younger. Additionally, patients who had been treated with methylprednisolone, or other immunomodulating agents were excluded from the present study. Finally, our control group consisted of symptomatic controls, not healthy controls, and an explanation for the unspecific symptoms in these individuals could be a general increased inflammatory activity.

In progressive courses of MS, absence of infiltration of inflammatory cells has been reported in several studies [8,9,32]. However, recently studies have shown that inflammation and neurodegeneration can be found in all lesions and stages of MS [33] and additionally, inflammatory biomarkers can be measured in the CSF from patients with progressive MS [34]. This is in agreement with our present findings of macrophage activity in PPMS similar to that of patients with a relapsing course of MS. Actually, both sCD163 CSF/serum ratio and index were high in PPMS in agreement with pathological investigations demonstrating continuous inflammation-mediated tissue injury [6]. It has also recently been shown that activated macrophages and microglia in active lesions express CD40 (M1 marker) while mannose receptor (MR) and CD163 (M2 markers) are expressed by myelin-laden macrophages in active lesions and perivascular macrophages. Double staining showed that approximately 70% of the CD40-positive macrophages in MS lesions also expressed MR, indicating that the majority of infiltrating macrophages and activated microglial cells display an intermediate activation status [9,12]. It could therefore be argued, that high levels of sCD163 in the CSF should be associated with high disability score as is indeed substantiated in the present study where there is a trend for a declivity in sCD163 levels with higher levels in PPMS, intermediate levels in RRMS and lower levels in CIS.

sCD163 is especially elevated in the patients with PPMS. This is particularly interesting since sCD163 may originate from M2 macrophages, notably involved in chronic inflammation. However, several factors could potentially contribute to the increased sCD163 levels in MS. It could be a consequence of an anti-inflammatory response, and mCD163 is strongly upregulated by IL-10 and glucocorticoid. It has been shown that $CD163^+$ macrophages are most abundant in acute active lesions and markedly decrease in number in chronic active lesions and further to chronic inactive lesions [8]. Furthermore, sCD163 has been shown to actively inhibit T-cell mediated immune reactions *in vitro*, thus exhibiting anti-inflammatory functions [26]. The close interactions between T cells and MHC class II^+ macrophages have also been reported in a study of parenchymal lesions in the spinal cord and meningeal lesions pointing towards a role in diffuse axonal loss [35]. This apposition of T cells and macrophages was seen in patients with progressive disease and could suggest antigen presentation and thus a mechanism for chronic inflammation. Even though the patients in the present study cohort have explicitly not been treated with glucocorticoids, it is possible that the local microenvironment could trigger a

similar reactivity [12,36]. One such trigger could be iron deposition which several studies have shown is increased in the plaques of patients with MS [37,38]. A recent study by Hametner et al [39] has shown an age-related increase of iron in the white matter of MS patients and this study suggests that the degeneration of cells in MS lesions leads to iron liberation from oligodendrocytes. To our knowledge there is no study of iron deposition related to CD163 expression in MS lesions but we suggest that a relationship is likely and that CSF sCD163 could be marker for this relationship. The PPMS patients have the highest mean age in our cohort and this corresponds well with the age-dependent increase of iron release in the white matter seen in the above mentioned study [39].The elevated levels of sCD163 could be a mechanism of tissue homeostasis and repair and thus CD163 could be a marker of immune modulatory functions regarding not only degeneration and anti-inflammation but also tissue repair [40,41].

The potential role of sCD163 in MS is presently unclear. sCD163 in CSF and serum was not correlated significantly to EDSS, duration of disease, time since last attack or number of attacks, but the sCD163 ratio was significantly increased in patients with MS/CIS. The membrane bound form of CD163 is up-regulated in MS lesions, and, as sCD163 is seen to be shed in other inflammatory conditions, we find that our results are highly suggestive of increased macrophage activation in patients with MS disease, particularly in patients with PPMS. These findings suggest that PPMS pathology is at least partly driven by inflammation. Previous studies [42,43] have shown that MS immune-modulating treatment, to some extent, have an effect on monocytes. Whether the level of sCD163 is affected by immune-modulating treatment and thus could function as a longitudinal disease activity marker for treatment efficacy has yet to be addressed.

The ROC analysis indicated the sCD163 CSF/serum ratio as only a fair diagnostic marker [44], and adding the results from sCD163 to that of IgG index (not shown) did not add further to the diagnostic properties of the IgG index.

In conclusion, soluble CD163 was measurable in CSF and serum from patients both with and without neurological deficits, and the sCD163 CSF/serum ratio was significantly increased in patients with inflammatory demyelinating disease. However, overlap of the levels of sCD163 between the groups and a ROC analysis that indicated sCD163 as only a fair marker makes the diagnostic utility of sCD163 limited. The sCD163 CSF/serum ratio was significantly increased in all groups of patients with MS and may reflect macrophage activation in MS lesions. These results suggest that primary progressive MS also is driven by inflammation in which the innate immune system plays a pivotal role.

Supporting Information

Data S1 File contains: Table S1 Table S1 includes Excel file and (double click to activate) contains all basic data for this paper. Abbreviations: RRMS (relapsing-remitting MS), PPMS (primary-progressive MS), SPMS (secondary-progressive MS), CIS (clinically isolated syndrome), SC (symptomatic controls with normal or abnormal MRI), n (number of persons), CSF (cerebrospinal fluid), y (years), d (days), Gender (1 = male; 2 = female), OND (other neurological disease), OMD (other medical disease). **Table S2** Table S2 lists proteins, their respective molecular weight and ratio CSF/serum. The table is adapted from Nockher et al (22). Abbreviations: kDA (kilo Dalton). **Table S3** Table S3 shows the output of our regression analysis on log transformed sCD163 serum values (lSerum) **Table S4** Table S4 shows the output of our regression analysis on log transformed sCD163 CSF values (lcsv). **Table S5** Table S5 shows the output of our regression analysis on log transformed scd163 CSF/serum values (lRatio). **Table S6** Table S6 shows the output of our regression analysis on log transformed sCD163 index values (lIndex) **Figure S1** Figure S1 shows the istribution of proteins and their CSF/serum ratios plotted against their molecular weight (in kD). This figure is adapted from Nockher et al (22). Proteins marked with (•) originate solely from the blood compartment (see appendix table 2 below) and proteins marked by (o) are suspected to be produced intrathecally.
(DOCX)

Acknowledgments

We thank laboratory technician Kirsten Bank Petersen for excellent work. We thank everybody at the MS clinic at the department of Neurology, Aarhus University Hospital. We thank associate professor Morten Frydenberg, Department of Public Health - Section of Biostatistics, Aarhus University who kindly assisted in the interpretation of our regression model.

Author Contributions

Conceived and designed the experiments: MS AKR TC HJM PVR TP. Performed the experiments: AKR MS HJM. Analyzed the data: MS HJM TP. Contributed reagents/materials/analysis tools: MS AKR HJM TC TP. Wrote the paper: MS AKR TC HJM PVR TP.

References

1. Compston A, Coles A (2002) Multiple sclerosis. Lancet 359: 1221–1231.
2. Compston A, Coles A (2008) Multiple sclerosis. Lancet 372: 1502–1517.
3. Kipp M, van der Valk P, Amor S (2012) Pathology of multiple sclerosis. CNS Neurol Disord Drug Targets 11: 506–517.
4. Lucchinetti C, Brück W, Parisi J, Scheithauer B, Rodriguez M, et al. (2000) Heterogeneity of multiple sclerosis lesions: implications for the pathogenesis of demyelination. Ann Neurol 47: 707–717.
5. Kornek B, Lassmann H (2003) Neuropathology of multiple sclerosis-new concepts. Brain Res Bull 61: 321–326.
6. Lassmann H, van Horssen J (2011) The molecular basis of neurodegeneration in multiple sclerosis. FEBS Lett 585: 3715–3723.
7. Mayo L, Quintana FJ, Weiner HL (2012) The innate immune system in demyelinating disease. Immunol Rev 248: 170–187.
8. Zhang Z, Zhang Z-Y, Schittenhelm J, Wu Y, Meyermann R, et al. (2011) Parenchymal accumulation of CD163+ macrophages/microglia in multiple sclerosis brains. J Neuroimmunol 237: 73–79.
9. Vogel DYS, Vereyken EJF, Glim JE, Heijnen PDAM, Moeton M, et al. (2013) Macrophages in inflammatory multiple sclerosis lesions have an intermediate activation status. J Neuroinflammation 10: 35.
10. Fabriek BO, Van Haastert ES, Galea I, Polfliet MMJ, Döpp ED, et al. (2005) CD163-positive perivascular macrophages in the human CNS express molecules for antigen recognition and presentation. Glia 51: 297–305.
11. Verreck FAW, de Boer T, Langenberg DML, van der Zanden L, Ottenhoff THM (2006) Phenotypic and functional profiling of human proinflammatory type-1 and anti-inflammatory type-2 macrophages in response to microbial antigens and IFN-gamma- and CD40L-mediated costimulation. J Leukoc Biol 79: 285–293.
12. Ambarus CA, Krausz S, van Eijk M, Hamann J, Radstake TRDJ, et al. (2012) Systematic validation of specific phenotypic markers for in vitro polarized human macrophages. J Immunol Methods 375: 196–206.
13. Durafourt BA, Moore CS, Zammit DA, Johnson TA, Zaguia F, et al. (2012) Comparison of polarization properties of human adult microglia and blood-derived macrophages. Glia 60: 717–727.
14. Galea J, Cruickshank G, Teeling JL, Boche D, Garland P, et al. (2012) The intrathecal CD163-haptoglobin-hemoglobin scavenging system in subarachnoid hemorrhage. J Neurochem 121: 785–792.
15. Davis BH, Zarev PV (2005) Human monocyte CD163 expression inversely correlates with soluble CD163 plasma levels. Cytometry B Clin Cytom 63: 16–22.
16. Fabriek BO, Moller HJ, Vloet RP, van Winsen LM, Hanemaaijer R, et al. (2007) Proteolytic shedding of the macrophage scavenger receptor CD163 in multiple sclerosis. J Neuroimmunol 187: 179–186.
17. Moller HJ, Hald K, Moestrup SK (2002) Characterization of an enzyme-linked immunosorbent assay for soluble CD163. Scand J Clin Lab Invest 62: 293–299.

18. Moller HJ (2012) Soluble CD163. Scand J Clin Lab Invest 72: 1–13.
19. Teunissen C, Menge T, Altintas A, Alvarez-Cermeno JC, Bertolotto A, et al. (2013) Consensus definitions and application guidelines for control groups in cerebrospinal fluid biomarker studies in multiple sclerosis. Mult Scler.
20. Polman CH, Reingold SC, Banwell B, Clanet M, Cohen JA, et al. (2011) Diagnostic criteria for multiple sclerosis: 2010 revisions to the McDonald criteria. Ann Neurol 69: 292–302.
21. Kurtzke JF (1983) Rating neurologic impairment in multiple sclerosis: an expanded disability status scale (EDSS). Neurology 33: 1444–1452.
22. Teunissen CE, Petzold A, Bennett JL, Berven FS, Brundin L, et al. (2009) A consensus protocol for the standardization of cerebrospinal fluid collection and biobanking. Neurology 73: 1914–1922.
23. R N (2003) Stata tip 1: The eform() option of regress. In: Newton HJ, editor. Stata Technical Journal. Stata Technical Journal: StataCorp LP.
24. Nockher WA, Wick M, Pfister HW (1999) Cerebrospinal fluid levels of soluble CD14 in inflammatory and non-inflammatory diseases of the CNS: upregulation during bacterial infections and viral meningitis. J Neuroimmunol 101: 161–169.
25. Fabriek BO, van Bruggen R, Deng DM, Ligtenberg AJM, Nazmi K, et al. (2009) The macrophage scavenger receptor CD163 functions as an innate immune sensor for bacteria. Blood 113: 887–892.
26. Högger P, Sorg C (2001) Soluble CD163 inhibits phorbol ester-induced lymphocyte proliferation. Biochem Biophys Res Commun 288: 841–843.
27. Brück W, Porada P, Poser S, Rieckmann P, Hanefeld F, et al. (1995) Monocyte/macrophage differentiation in early multiple sclerosis lesions. Ann Neurol 38: 788–796.
28. Moller HJ, Aerts H, Gronbaek H, Peterslund NA, Hyltoft Petersen P, et al. (2002) Soluble CD163: a marker molecule for monocyte/macrophage activity in disease. Scand J Clin Lab Invest Suppl 237: 29–33.
29. Moller HJ, de Fost M, Aerts H, Hollak C, Moestrup SK (2004) Plasma level of the macrophage-derived soluble CD163 is increased and positively correlates with severity in Gaucher's disease. Eur J Haematol 72: 135–139.
30. Baeten D, Moller HJ, Delanghe J, Veys EM, Moestrup SK, et al. (2004) Association of CD163+ macrophages and local production of soluble CD163 with decreased lymphocyte activation in spondylarthropathy synovitis. Arthritis Rheum 50: 1611–1623.
31. Hiraoka A, Horiike N, Akbar SM, Michitaka K, Matsuyama T, et al. (2005) Soluble CD163 in patients with liver diseases: very high levels of soluble CD163 in patients with fulminant hepatic failure. J Gastroenterol 40: 52–56.
32. Trapp BD, Nave K-A (2008) Multiple sclerosis: an immune or neurodegenerative disorder? Annu Rev Neurosci 31: 247–269.
33. Frischer JM, Bramow S, Dal-Bianco A, Lucchinetti CF, Rauschka H, et al. (2009) The relation between inflammation and neurodegeneration in multiple sclerosis brains. Brain 132: 1175–1189.
34. Romme Christensen J, Börnsen L, Khademi M, Olsson T, Jensen PE, et al. (2013) CSF inflammation and axonal damage are increased and correlate in progressive multiple sclerosis. Mult Scler 19: 877–884.
35. Androdias G, Reynolds R, Chanal M, Ritleng C, Confavreux C, et al. (2010) Meningeal T cells associate with diffuse axonal loss in multiple sclerosis spinal cords. Ann Neurol 68: 465–476.
36. Timmermann M, Buck F, Sorg C, Högger P (2004) Interaction of soluble CD163 with activated T lymphocytes involves its association with non-muscle myosin heavy chain type A. Immunol Cell Biol 82: 479–487.
37. Adams CW (1988) Perivascular iron deposition and other vascular damage in multiple sclerosis. J Neurol Neurosurg Psychiatry 51: 260–265.
38. Mehta V, Pei W, Yang G, Li S, Swamy E, et al. (2013) Iron is a sensitive biomarker for inflammation in multiple sclerosis lesions. PLoS One 8: e57573.
39. Hametner S, Wimmer I, Haider L, Pfeifenbring S, Brück W, et al. (2013) Iron and neurodegeneration in the multiple sclerosis brain. Ann Neurol.
40. Ellwardt E, Zipp F (2014) Molecular mechanisms linking neuroinflammation and neurodegeneration in MS. Exp Neurol.
41. Mosser DM, Edwards JP (2008) Exploring the full spectrum of macrophage activation. Nat Rev Immunol 8: 958–969.
42. Skarica M, Eckstein C, Whartenby KA, Calabresi PA (2011) Novel mechanisms of immune modulation of natalizumab in multiple sclerosis patients. J Neuroimmunol 235: 70–76.
43. Carpintero R, Brandt KJ, Gruaz L, Molnarfi N, Lalive PH, et al. (2010) Glatiramer acetate triggers PI3Kδ/Akt and MEK/ERK pathways to induce IL-1 receptor antagonist in human monocytes. Proc Natl Acad Sci U S A 107: 17692–17697.
44. Xia J, Broadhurst DI, Wilson M, Wishart DS (2013) Translational biomarker discovery in clinical metabolomics: an introductory tutorial. Metabolomics 9: 280–299.
